# Sustainable Fabrication of Tailored Bone Substitutes: From High‐Throughput Scaffold Manufacturing, Scaled‐Up HMSC Expansion to Dynamic Cultivation in a Perfusion Bioreactor

**DOI:** 10.1002/advs.202523846

**Published:** 2026-07-23

**Authors:** Franziska Braun, Anna Paříková, Sandra Rother, Martin Kantor, Salman Muhammad Ilyas, Ricardo Bernhardt, Pavel Ndjawa Yomi, Jaromír Havlica, Revathi Appali, Benjamin Kruppke, Poh Soo Lee

**Affiliations:** ^1^ Institute of Materials Science Max Bergmann Center of Biomaterials TUD Dresden University of Technology Dresden Germany; ^2^ Institute of Chemical Process Fundamentals The Czech Academy of Sciences Prague Czech Republic; ^3^ Department of Chemistry Jan Evangelista Purkyně University in Ústí nad Labem Ústí nad Labem Czech Republic; ^4^ Institute of Biophysics Center of Integrative Physiology and Molecular Medicine (CIPMM) Saarland University Homburg Germany; ^5^ Institute of Machines and Power Engineering Faculty of Mechanical Engineering University J.E. Purkyně Ústí nad Labem Czech Republic; ^6^ Department Materials Engineering Institute of Polymers Materials Leibniz‐Institut Für Polymerforschung Dresden e.V. (IPF) Dresden Germany; ^7^ Institute of General Electrical Engineering Faculty of Computer Science and Electrical Engineering University of Rostock Rostock Germany; ^8^ Department of Ageing of Individuals and Society Interdisciplinary Faculty University of Rostock Rostock Germany; ^9^ Institute For Organic and Macromolecular Chemistry Friedrich Schiller University Jena Jena Germany; ^10^ Department of Biomaterials Institut Für Bioprozess‐ und Analysenmesstechnik e.V. Heilbad Heiligenstadt Germany

**Keywords:** 3D‐printing, biomimetic bioreactor, endochondral ossification, human mesenchymal stromal cells, modular manufacturing, oxygen tension, spinner flask

## Abstract

The demand for off‐the‐shelf biocompatible bone substitutes has driven the development of numerous independent in vitro technologies to generate products resembling physiological tissues. Due to technical challenges and overly simplified cultivation approaches/niches, the end‐products are often uniformly shaped and inferior to native bone tissue. In this report, three major technologies are implemented cohesively to address these shortfalls: (1) Spinner flasks for scaled‐up stem cell expansion, (2) Manufacturing via 3D‐printing and cast‐molding processes, large modular collagen‐based (COL) scaffolds +/− chondroitin sulfate A (CSA) of tailored dimensions, (3) Perfusion bioreactor with controlled oxygen tension (pO_2_) to support high cell density and osteochondral differentiation. We report an oxygenated in vitro niche within the bioreactor that supports high cell density and self‐induced osteogenic differentiation for 60 days. Enhanced mineralization and osteogenic gene expression in COL scaffolds were observed, while COL + CSA scaffolds exhibited elevated *Col10a* gene expression for hypertrophic chondrocytes, a representative indicator of active endochondral ossification. In summary, this report describes an integral approach to consistently and rapidly achieve physiologically relevant bone substitutes of tailored dimensions. Furthermore, the pivotal effect of recapitulating endochondral ossification in vitro through dynamic bioreactor culture is demonstrated, paving the way to generate complex tissue structures in the future.

## 1 Introduction

The enormous need for off‐the‐shelf bone substitutes has driven the development of multiple technologies to generate large, engineered bone substitutes and/or support their continual propagation in vitro to achieve end‐products that possess mechanical and physical properties resembling the native bone tissues [1, 2]. For example, devices for additive manufacturing are frequently used in tissue engineering applications due to their capability to generate sizable, freeform bone constructs in a controlled, precise manner. This enabled rapid prototyping with polymers for experimental trials and deriving tailored constructs of variable shapes and sizes [3, 4]. However, technical limitations have hindered the generation of complex collagen‐based scaffolds using a 3D printer [5]. A major challenge is the insufficient structural stability of collagen, which frequently leads to the sample collapsing during printing, particularly as scaffold size increases or when printing irregular dimensions. Otherwise, a significant amount of supporting materials/structures deemed redundant after printing must be included [5]. Nevertheless, collagen‐based scaffolds are more biologically relevant and often preferred in tissue engineering for their potential to support cellular infiltration, osseointegration, and reduced immunological responses [5, 6]. Therefore, alternative approaches that further leverage the advantages of 3D printing to produce consistent, large collagen‐based scaffolds with tailored dimensions and high structural stability are necessary. Moreover, the 3R principles (Replacement, Reduction, and Refinement) for sustainability in research should be extended to the fabrication process to minimize material waste and maximize manufacturing efficiency [3, 7, 8].

Besides scaffold fabrication, achieving high cell density and maintaining a diverse osteochondral cell population in the engineered bone constructs are equally critical. Stem cells are widely used as a source of cells in tissue engineering applications, mainly because of their self‐renewal capacity to generate more cells and their potential to differentiate into multiple cell types [9]. However, deriving large quantities of undifferentiated stem cells with conventional cell culture vessels is labor‐intensive and time‐consuming. For this purpose, spinner flasks have been developed to scale up stem cell expansion and efficiently derive undifferentiated stem cells in bulk in a shorter period of time [10, 11, 12, 13]. In the case of anchorage‐dependent human bone marrow‐derived mesenchymal stromal cells (hMSCs), microcarrier beads with increased potential surface area are used [10, 12, 13]. For human embryonic and induced pluripotent stem cells (hiPSC) [11], direct expansion as embryoid bodies is done. Furthermore, adequate fluid shear stress generated in spinner flasks has been shown to increase stem cell proliferation while retaining pluripotency [10, 11, 12, 13]. Although proven effective, this technology has not been implemented consistently because most research studies use either low cell densities in their scaffolds or small scaffold geometries, thereby ensuring adequate oxygen and nutrient supply [14, 15]. This is often due to the limitations of conventional in vitro cell culture approaches in flasks/well‐plates, as the static niche hinders efficient mass transfer [16, 17]. Fluctuations in nutrient distribution and oxygen tension often lead to heterogeneity in stem cell differentiation or induce accelerated cell death in scaffolds with high cell density. Consequently, there are significant cellular, mechanical, and physical variabilities in different parts of an engineered bone substitute cultured in vitro [16, 17, 18, 19]. As a solution, various perfusion bioreactor designs have been developed to support the stable in vitro propagation of large 3D bone substitutes at high cell density [18, 19, 20].

Unlike conventional cell culture vessels, the perfusing medium in a bioreactor enhances mass transfer and oxygen distribution within bone substitutes [19, 20, 21, 22, 23]. The resulting fluid shear stress from the perfusing medium also enhanced hMSC osteogenic differentiation and mineralization [19, 20, 23]. However, limitations remain in the cultivation approaches used in these bioreactors, which generally require a larger volume of differentiation medium supplemented with costly growth factors. First, the proficiency of exogenous growth factors to penetrate the engineered constructs decreases with increasing size. Although this can be improved by increasing the medium perfusion rate, the excessive fluid shear stress can cause structural damage. Second, the effects of growth factors are rather specific and favor a particular cell type. As a result, engineered bone constructs often lack the diverse cell populations found in native bone tissues. Therefore, further exploiting bioreactor specifications, such as perfusion rate and partial oxygen tension (pO_2_), to generate dynamic cultivation niches will be a logical strategy for further advancements. These should initiate cues for osteochondral ossification and be self‐regulated by cells within the bone construct. This will not only yield a diverse osteochondral cell population, but the biomimetic niches will also reduce the need for exogenous growth factor supplementation to induce stem cell differentiation [23, 24, 25]. Specifically, localized cell–cell and cell–matrix interactions should trigger the self‐production of essential growth factors necessary for osteogenesis [23, 25].

In this study, the 3D printer, spinner flask, and perfusion bioreactor technologies are coupled in series. A conceptual approach is implemented to enable the flow production of large, tailored, off‐the‐shelf bone substitutes. Specifically, large, lyophilized type 1 collagen (COL) scaffolds with tailored dimensions and a defined Young´s modulus are produced in bulk via 3D printing and cast‐molding. Further, chondroitin sulfate A (CSA), a potent glycosaminoglycan that induces chondrogenic differentiation [23, 26], and also a key component of cartilage tissue with anti‐inflammatory/chondroprotective effects, is included [27, 28]. Specifically for CSA subtype, also known as chondroitin‐4‐sulfate, contains d‐glucuronic acid and *N*‐galactosamine sulfated at the C4 position [28, 29]. It is found in the proteoglycans decorin and biglycan, present not only in the cartilage but also in bone tissue [28, 29]. Within the context of this study, CSA is embedded within the collagen‐based scaffold to substitute conventional chondrogenic growth factors, for example, transforming growth factor‐beta (TGF‐β), bone morphogenetic proteins (BMP), insulin‐like growth factors (IGF), fibroblast growth factors (FGF) [30]. The objective is to recapitulate endochondral ossification within the bone construct, the underlying process for long‐bone development and fracture healing [31, 32]. To further strengthen the physiological relevance and achieve the diverse osteochondral population resembling native bone tissue, hMSCs generated in a spinner flask were inoculated into the COL scaffold at high density (up to 3.5 × 10^6^ cells). Specifically, the proficiency in achieving different osteochondral cell types—such as chondrocytes, hypertrophic chondrocytes, osteoprogenitors, and osteoblasts—is assessed through gene marker expression profile. To support the continual propagation of these scaffolds densely seeded with hMSC, they are cultured in a previously established perfusion bioreactor with a defined pO_2_ distribution [20, 23]. Similar to the physiological conditions, hMSC differentiation is initiated locally within the bioreactor through cell–cell interactions and biochemical/physical cues. Thereby eliminating the need for exogenous growth factor supplements, such as TGF‐β, BMP, IGF, and FGF [33].

## 2 Results

### 2.1 Effective Upscaling hMSC Expansion in Spinner Flasks and CFD Modeling

The computational fluid dynamics (CFD) model in Figure 1A indicates a linear correlation between shear rate (s^−1^) and stir rate (rpm), and the average shear‐strain schematic at 90 rpm (Figure 1C) shows homogeneous fluid shear throughout. The simulations at 60 and 120 rpm across different planes are shown in Figure S1. Furthermore, the calculated maximum shear stress (dyne/cm^2^) summarized in Figure 1B was also close to the optimized condition previously defined in a 125 mL flask [12, 13]. Therefore, a stirring rate of 90 rpm was applied for hMSC expansion in this study. After 12 days of cell expansion with an initial hMSC density of 2 × 10^6^, an average of 19.28 ± 2.46 × 10^6^ hMSC was harvested from a single spinner flask, while only 7.84 ± 0.58 × 10^6^ hMSC was harvested from two T175 flasks. These values were derived from viable cell counts of trypan‐blue‐stained cell suspension using an automatic cell counter. A total of 3 hMSC donors were investigated in triplicate, and the means of each donor are shown in Figure S2A, coupled with a lactate dehydrogenase (LDH) cytotoxicity assay. Notably, a donor effect was observed in the LDH assay, with lower cell counts for donor #2 compared with donors #1 and #3, which were similar. The morphology of hMSC from the spinner flask and T175 was similar after 1 day post‐expansion, and the typical spindle‐like morphology of hMSC was documented in both cases (Figure 1D, 10× magnification at 1 day). After 6 days of expansion in a 6‐well plate, the hMSC from both setups reached 90% confluency (Figure 1D). Upon isolation of primary cells from the iliac crest of healthy donors (See Section 5.1), the cells were sorted with flow cytometry to select for hMSCs, using standard hMSC markers. Based on this, the relative gene expression of *CD90* and *Sox2*, two characteristic hMSC markers, is used to facilitate rapid assessment of hMSC stemness in this study after cell expansion in T175/spinner flasks. Generally, the hMSCs from both sources have similarly higher relative gene expression than the original undifferentiated hMSCs from d0 (Figure 1E). This was further validated by standard trilineage differentiation assays (Figure 1F), which showed that hMSCs from both sources have retained their multipotency and that those from the spinner flask exhibited a more pronounced trilineage differentiation. Specifically, for chondrogenic differentiation, which resulted in more condensed aggregates (indicated by the arrows) and more positively stained adipogenic differentiated cells (adi). Despite the presence of additional shear rate in the spinner flask, the hMSC expanded in the spinner flask does not show a significant deterioration in stemness and multipotency, which are also comparable to the undifferentiated hMSC and the conventional T175‐derived hMSC.

**FIGURE 1:**
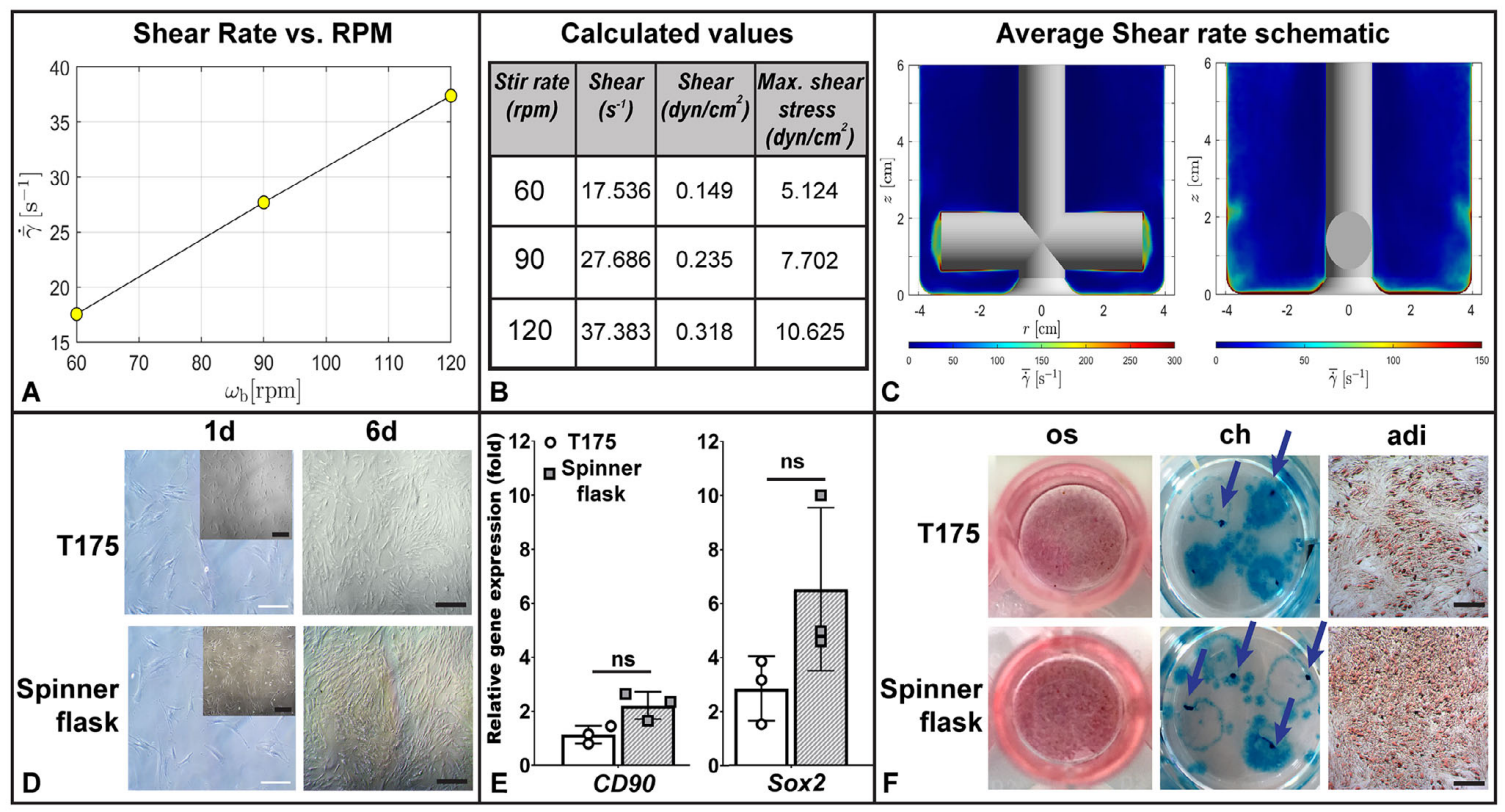
Shear‐rate modeling for spinner flasks and comparing hMSCs expanded in a spinner flask versus a T175 cell culture flask. (A) Linear correlation of shear strain rate against stir rate (rpm). (B) Calculated shear stress in the spinner flask at 60, 90, and 120 rpm. (C) Computational fluid dynamics (CFD) simulation of the spinner flask at the optimum stirring rate (90 rpm), showing the spatial distribution of the average shear rate. (D) hMSCs expanded on a T175 flask and a spinner flask for 12 days possessed the typical spindle‐like cell morphology after passaging and were expanded on a 6‐well plate for 1 day. This is a representative image of three hMSC donors and the experimental setup in triplicate; the cell morphology remained unchanged after 6 days, as the cell culture reached 90% confluency (*n* = 3). Scale bar: 100 µm (black), 200 µm (white). (E) The relative gene expression of hMSC markers, *CD90* and *Sox2*, after 12 days of cell expansion; Student's *t*‐test with no statistical significance (ns). The hMSC from each source was normalized against the original undifferentiated hMSCs. The qPCR was performed in triplicate, and the mean values are tabulated (*n* = 3). (F) Standard osteogenic (os), chondrogenic (ch), and adipogenic (adi) differentiation protocol to validate the multipotency of hMSC expanded in a spinner flask and T175 flask. The arrows mark the condensed cell spheroids for positive chondrogenic differentiation. This is a representative image for all three hMSC donors (*n* = 3), and each differentiation test was performed in triplicate.

### 2.2 Fabricating Structurally Stable Collagen‐Based Scaffolds With Tailored Dimensions

By coupling 3D printing with a cast‐molding approach described in Section 5.5, structured collagen‐based scaffolds of centimeter‐scale with tailored dimensions were fabricated (Figure 2, left panel). Based on the scale in Figure 2, the calculated mean volume for each section was—top section + top 5 mm cap: 0.25 cm^3^; top section only: 0.19 cm^3^; bottom section: 0.10 cm^3^. Since the top 5 mm cap was in direct contact with the agarose in the bioreactor, it was omitted from most analyses for accurate interpretation, except for absolute cell number and pO_2_ measurements, where the entire scaffold is critical.

**FIGURE 2:**
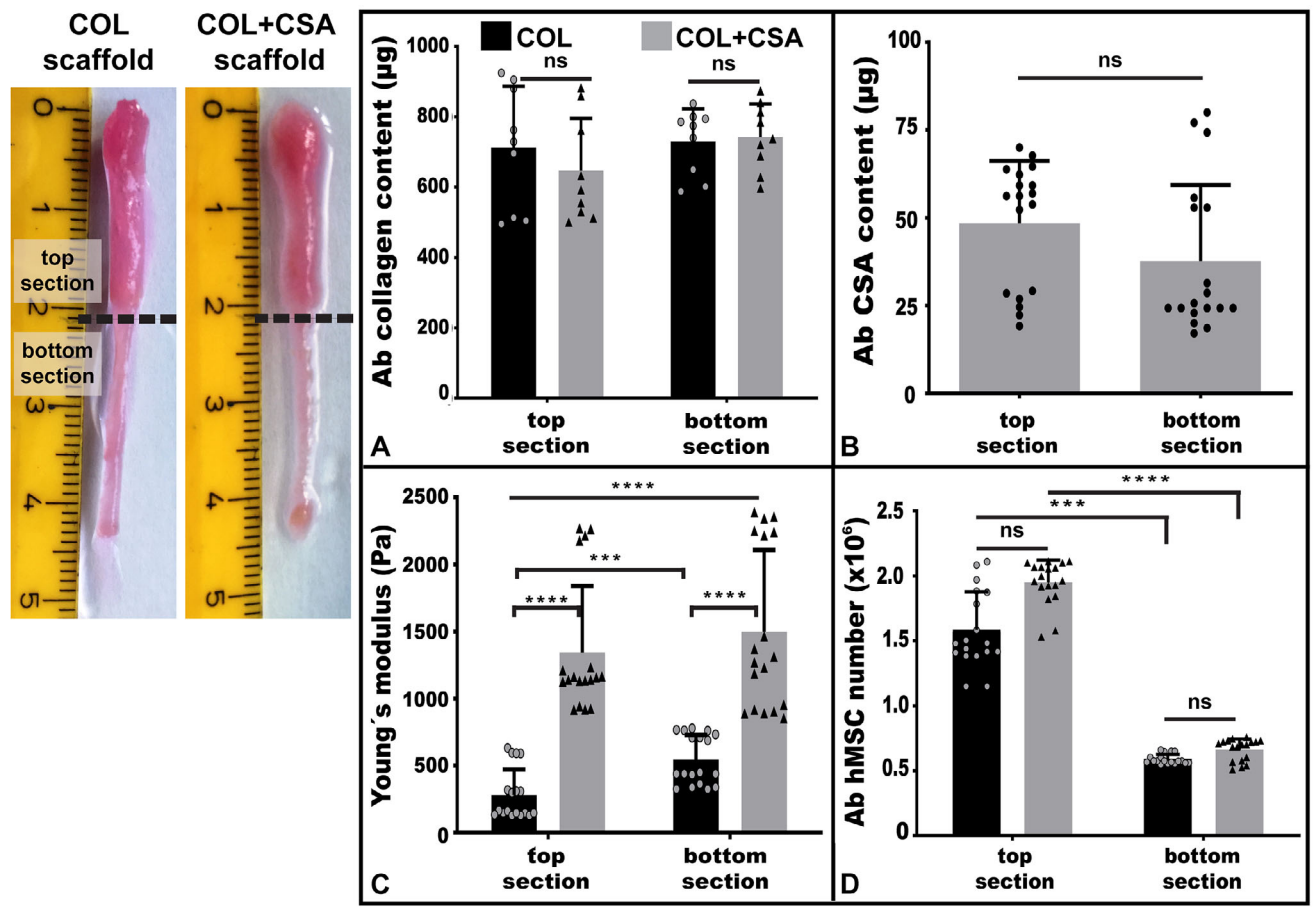
Composition and properties of Collagen (COL) and COL + Chondroitin Sulfate A (CSA) scaffolds. (Left) Physical appearance of COL and COL + CSA scaffolds after equilibration in serum‐free DMEM medium for 1 day; representative image of cell‐free scaffolds shown in Figure 4C (*n* = 3, 3 independent batches). (A) Absolute collagen content in the respective scaffold. (B) Absolute CSA amount in each section of COL + CSA scaffolds (*n *= 6, 3 independent batches). (C) Young´s modulus of initial COL and COL + CSA scaffolds (*n *= 6, 3 independent batches), (D) Absolute number of hMSCs in each section of a scaffold 1 day post‐inoculation (*n *= 3, triplicates from 2 independent experiments). Student *t*‐test, ns = no significance, *** *p* < 0.001, **** *p* < 0.0001.

Despite differences in dimensions/volume, the absolute collagen‐to‐CSA content in the top and bottom sections of these tailored COL and COL + CSA scaffolds was similar and comparable (Figure 2A,B). The mean absolute collagen content was—COL top section (0.19 cm^3^): 699.8 ± 169.1 µg, COL bottom section (0.10 cm^3^): 747.9 ± 81.1 µg, COL + CSA top section (0.19 cm^3^): 644.7 ± 140.9 µg, and COL + CSA bottom section (0.10 cm^3^): 756.4 ± 89.9 µg. The variances between the COL and COL + CSA scaffolds were not significant. The mean CSA content in COL + CSA scaffolds was 48.5 ± 17.7 µg in the top section and 37.7 ± 21.6 µg in the bottom section. In terms of Young´s modulus (Figure 2C), the bottom sections of both COL and COL + CSA scaffolds are significantly stiffer than the corresponding top sections. The mean Young´s modulus of the COL bottom section was 1354.2 ± 510.2 Pa, and COL + CSA was 1445.9 ± 592.1 Pa, with no significant variance (*p* = 0.65). In contrast, the top section of COL scaffolds was 270.1 ± 182.5 Pa, while COL + CSA was 511.6 ± 230.8 Pa, with significant variance (*p* = 0.0003). The significant impacts of scaffold geometry/design on both factors were demonstrated.

### 2.3 Proficiency of hMSC Inoculation on Scaffolds, Cell Proliferation and Oxygen Tension (pO_2_)

The mean absolute cell number in the respective bone construct was COL top (0.25 cm^3^): 1.63 ± 0.29 × 10^6^, COL bottom (0.10 cm^3^): 0.59 ± 0.04 × 10^6^, COL + CSA top (0.25 cm^3^): 1.92 ± 0.17 × 10^6^, COL + CSA bottom (0.10 cm^3^): 0.65 ± 0.09 × 10^6^ (Figure 2D). With the initial inoculation density of 3.5 × 10^6^ hMSCs applied to each scaffold, the overall proficiency of hMSC inoculation using the agarose wells described in section 5.6 was 63.4% for COL scaffolds and 73.4% for COL + CSA scaffolds.

#### 2.3.1 Evaluating the Cell Number in Samples of Different Geometries Over Time

The absolute cell number in bone constructs retrieved after 30 and 60 days is presented in Figure 3A and determined through DNA concentration. Due to inconsistent geometries/condensation of different bone constructs over time, the cell density per cm^3^ could not be determined. Briefly, the overall cell number in both COL and COL + CSA bone constructs from static/perfusion cultures was less than the initial seeding density. Specifically for perfusion culture in 30 days, but showed higher cell numbers by 60 days. With a larger volume, significantly more cells were present in the top sections of both bone construct variants. For the bottom sections, the cell numbers correspond to the extent of condensation shown in Figure 4. Specifically for COL 60 days static (Figure 3A), the severe shrinkage of the bone construct resulted in fewer cells in the bottom section. The cell number was also evaluated using the lactate dehydrogenase (LDH) cell viability assay and is shown in Figure S2B. As with DNA concentration, a similar trend is observed in each section of the bone construct cultured under static versus perfusion conditions at 30 and 60 days, but the LDH assay showed a higher cell number. Furthermore, a larger standard deviation, especially at 60 days, is reported. This is generally due to different hMSC donors.

**FIGURE 3:**
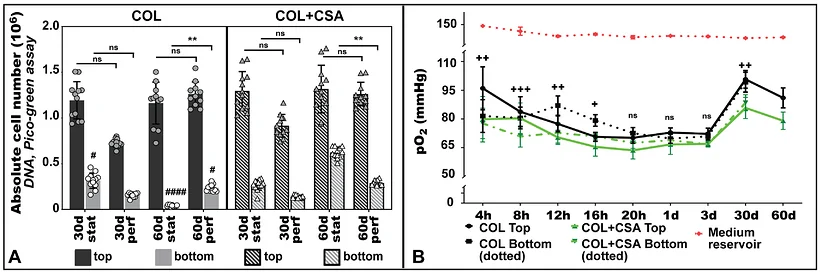
Cell proliferation, partial oxygen tension in bone constructs over 60 days. (A) Pico‐green (DNA) assay for determining the absolute cell number in top/bottom sections of COL and COL + CSA bone constructs cultured 30 and 60 days at static / perfusion (perf) state. (*n *= 3, duplicates, 2 independent experiments), Two‐way ANOVA and Tukey multiple *t*‐tests. * *p* < 0.05, ** *p* < 0.01, *** *p* < 0.001, **** *p* < 0.0001. (B) Partial oxygen tension (pO_2_) in the top/bottom sections of COL and COL + CSA bone constructs until 60 days. Due to the small diameter of the bottom section at 60 days and the slippage of the pO_2_ fiber optic probe, no measurements could be made. Student *t*‐test was applied to determine variance between samples at the respective time points (*n *= 3); ns = no significance, **^+^** *p* < 0.05, **^++^** *p* < 0.01, **^+++^** *p* < 0.001, **^++++^** *p* < 0.0001.

**FIGURE 4:**
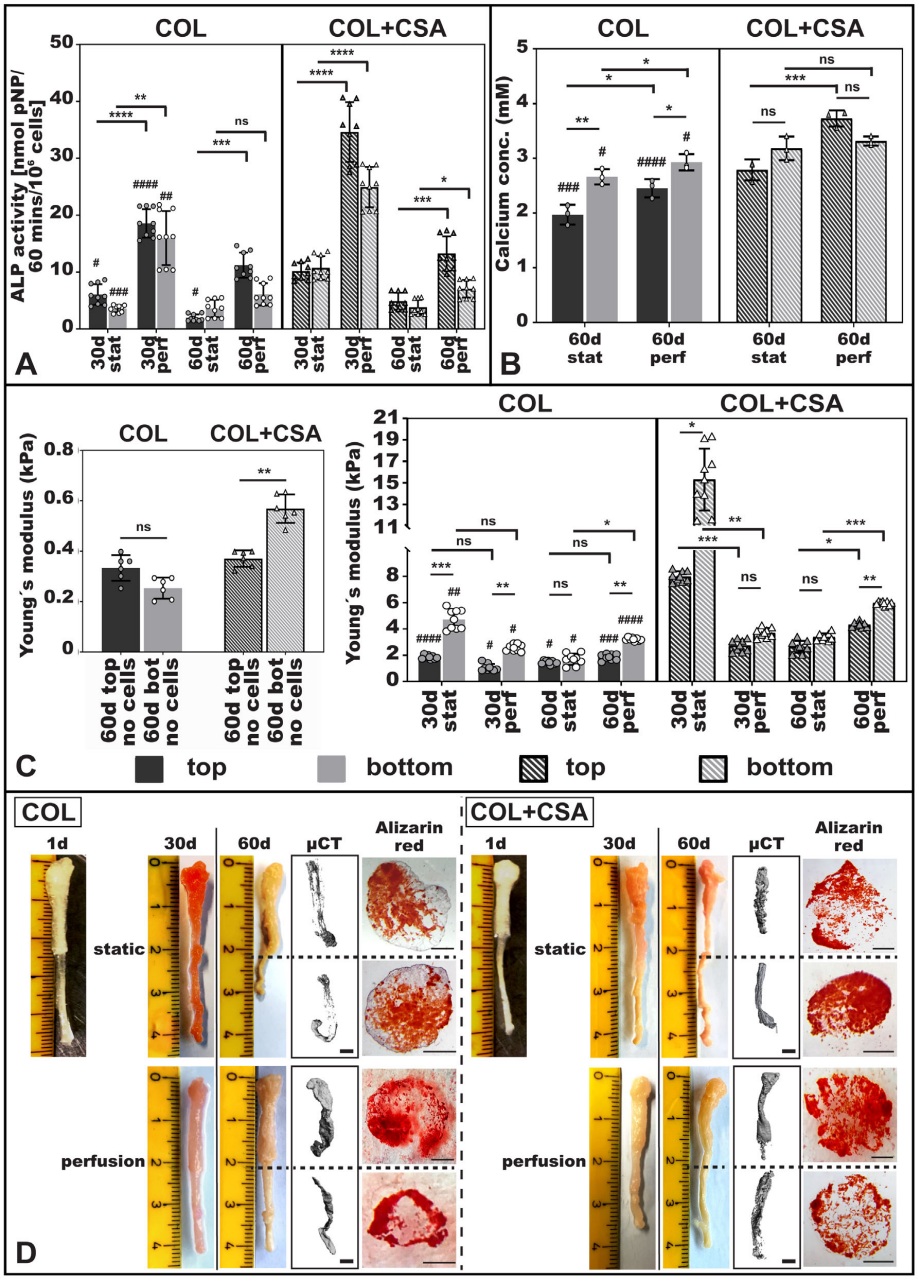
Physical characteristics, osteogenic differentiation, and mineralization of bone constructs derived from different cultivation conditions. (A) Alkaline phosphatase (ALP) activity, an indicator for initiation of osteogenic differentiation, in respective samples on 30 and 60 days. Two‐way ANOVA and Tukey multiple *t*‐test comparison (*n* = 3, 3 independent experiments) were applied for statistical analysis. (B) Concentration of calcium deposits accumulated within individual samples after 60 days. Two‐way ANOVA and Tukey multiple *t*‐test comparison (*n* = 3) were applied for statistical analysis. (C) The Young´s modulus of individual samples (left) cell‐free scaffolds incubated in os medium for 60 days, (right) hMSC‐laden bone constructs after 30 and 60 days. Two‐way ANOVA and Tukey multiple *t*‐test comparison (*n* = 3, 2 independent experiments for samples with no cells, left; 3 independent experiments for samples with cells, right) were applied for statistical analysis. The physical appearances of constructs after 1, 30, and 60 days of cultivation in the perfusion bioreactor and their corresponding static culture. Additionally, µCT was performed on 60‐day bone constructs to assess the overall mineralization state of each construct (scale bar: 2 mm). Alizarin red staining was performed on the cross‐section of each construct to validate calcium accumulation (scale bar: 1 mm). * *p* < 0.05, ** *p* < 0.01, *** *p* < 0.001, **** *p* < 0.0001. # indicates the variance of COL and the corresponding COL + CSA sample. # *p* < 0.05, ## *p* < 0.01, ### *p* < 0.001, #### *p* < 0.0001.

#### 2.3.2 Partial Oxygen Pressure (pO_2_)

Point‐specific measurements were performed to monitor the pO_2_ at the top, bottom section, and medium reservoir over 60 days (Figure 3B). No measurements could be taken from the bone constructs in static culture, as they were submerged at the bottom of the T75 flask. Unlike the perfusion bioreactor, there was no point of support to secure the fiber‐optic probe, ensuring the same position on the bone construct was measured. Moreover, the static condition does not support the extensive mass transfer that the perfusion bioreactor is designed to enable. The pO_2_ in the medium reservoir (red line) remained consistent throughout 60 days, at 137.5 ± 4.9 mmHg. It was also shown that the bioreactors reached an equilibrium state after 16 h of medium perfusion at 5.5 mL/min, and no statistically significant differences were observed between the top/bottom sections and between COL/COL + CSA samples until after 3 days. With the vast variance in absolute cell number indicated in Figure 3A, a significant variance of pO_2_ in the top and bottom sections of the bone construct would be expected. Nevertheless, this was not the case. Therefore, the pO_2_ was correlated to the calculated cell densities in respective sections—COL top (0.25 cm^3^): 6.52 ± 1.16 × 10^6^ hMSC/cm^3^, COL bottom (0.10 cm^3^): 5.91 ± 0.44 × 10^6^ hMSC/cm^3^, COL + CSA top (0.25 cm^3^): 7.68 ± 0.68 × 10^6^ hMSC/cm^3^, COL + CSA bottom (0.10 cm^3^): 6.51 ± 0.93 × 10^6^ hMSC/cm^3^. These calculated values, with narrower gaps between the top and bottom sections, indicated that cell density (cells/cm^3^) was the determinant of measured pO_2_ in the perfusion bioreactor. After 30 days of culture, an increase in pO_2_ was documented, which correlated with fewer cells than the initially seeded cells (Figure 3A). With continued cell proliferation until 60 days, pO_2_ declined again, reflecting increased oxygen consumption by proliferating cells. It should also be noted that pO_2_ measurements in the bottom section were not possible on 60 days, due to the smaller diameter of the bone constructs and technical difficulties in preventing the pO_2_ sensor from slipping out of the scaffold. In summary, Figure 3B shows that the bone constructs were consistently maintained within an oxygenated niche when a medium perfusion rate of 5.5 mL/min was applied through the bioreactor.

### 2.4 Bone Constructs From the Perfusion Bioreactor Versus Static Culture and Osteogenic Characteristics

#### 2.4.1 ALP Activity and Calcium Deposition

The increase in ALP activity generally marks the beginning of hMSC osteogenic differentiation to osteoblasts and is commonly used as an early indicator. Interestingly, a significantly elevated ALP activity was still observed in both COL and COL + CSA bone constructs cultured in the perfusion bioreactor for 30 days, with the latter being superior (Figure 4A). Although the top section of COL + CSA possessed a higher ALP activity, it should be noted that the bottom section had a lower number of cells (Figure 3A). By 60 days, the ALP activities had subsided. Generally, the perfusion culture also showed a higher ALP activity than the corresponding static culture until 60 days.

As part of the endpoint evaluation, the total calcium concentration in each sample was quantified using the Fluitest CA CPC kit. This is done to provide a quantitative assessment of calcium deposition and evaluate construct mineralization over time (Figure 4B). Since accumulated mineral takes time to be visible in alizarin red staining and µCT analysis, only the results from 60 days are presented. Interestingly, the bottom section (0.10 cm^3^) generally has a higher calcium concentration than the top section (0.25 cm^3^), despite a smaller initial volume in design and severe shrinkage observed in the static culture (Figure 4D). Nevertheless, there is little/no significant variance between static and perfusion culture. An exception is the top section of COL + CSA perfusion culture, which showed a significantly higher amount of calcium deposits than the corresponding static culture, indicative of the enhancing effects of perfusion in scaffolds with greater volume.

#### 2.4.2 Young's Modulus of Bone Constructs Over Time

The Young´s modulus measurements were performed to elucidate the contribution of calcium deposition to scaffold stiffness (Figure 4C). In comparison to the Young´s modulus of scaffolds from the initial timepoint (Figure 2C), the Young´s modulus of cell‐free scaffolds after incubation in os medium for 60 days was reduced by at least 25% (Figure 4C, left), especially for the COL scaffolds, an indication of scaffold degradation. Similar to the initial state, the bottom section of COL + CSA remained stiffer than the top section. Overall, the COL + CSA also had a higher Young´s modulus than the corresponding COL, and the bottom sections were also slightly stiffer than the top sections, a probable effect caused by scaffold geometries and volume (Figure 4D). Interestingly, COL + CSA static culture showed the most significant changes in Young´s modulus, with the highest stiffness at 30 d but a significant decrease at 60 days, an effect due to the reduced diameter. Otherwise, the bone constructs cultured under other conditions followed a similar trend, with a progressive increase in Young´s modulus until 60 days. Specifically, the perfusion cultures from 60 days were also superior to the corresponding static cultures.

#### 2.4.3 The Development and Mineralization of COL and COL + CSA Bone Constructs Over 60 days

Figure 4D summarizes the gross physical appearances and the development of bone constructs retrieved from a perfusion bioreactor, and the corresponding static cultures at 1, 30, and 60 days. Compared to the 1d constructs, which had a translucent texture, all other bone constructs exhibited an ivory coloration and a denser texture. Furthermore, these changes became more pronounced with extended cultivation periods. Regarding structural integrity, the bone constructs derived from static cultures exhibited significant condensation and shrinkage, particularly the COL constructs from 60 days. In contrast, the structural integrity of both COL and COL + CSA bone constructs cultivated in the perfusion bioreactor for 60 days remained intact, and their dimensions were comparable to those of the original scaffold at 1 day.

Additionally, the µCT images (Figure 4D) showed higher and more homogeneous mineralization throughout the scaffolds from the perfusion bioreactor than from static cultures, indicating that medium perfusion enhances construct mineralization. From the µCT images of 60‐day samples, the mineral ratio (MV/TV) was used to calculate the density (mg/cm^3^) using a hydroxyapatite phantom as a calibrator (Table 1). Other than the COL + CSA perfusion construct, the bottom section of all other samples possesses a higher density than the corresponding top section. Overall, the calculated density can be correlated to the calcium assay, including the COL + CSA perfusion samples, indicating a consistent trend in mineralization of bone constructs.

**TABLE 1:** Calculated density of 60 days samples based on mineral ratio (MV/TV) against hydroxyapatite phantom from µCT image.

Samples	Calculated density	Samples	Calculated density
COL static top	160 mg/cm^3^	COL + CSA static top	92.2 mg/cm^3^
COL static bot	183 mg/cm^3^	COL + CSA static bot	115 mg/cm^3^
COL perfusion top	173 mg/cm^3^	COL + CSA perfusion top	161.6 mg/cm^3^
COL perfusion bot	175 mg/cm^3^	COL + CSA perfusion bot	150.6 mg/cm^3^

In addition to µCT imaging, qualitative analyses with alizarin red staining were done to locate and visualize the mineralized regions in each bone construct and to confirm that the µCT images were not artifacts. Generally, homogeneous alizarin red staining was observed in the bottom section of COL + CSA static cultures, indicating mineralization in these bone constructs. Interestingly, CSA enhanced the structural integrity of perfusion cultures, as evidenced by both µCT images and alizarin red staining. In COL constructs, µCT images revealed a collapsed structure with a thin, compact mineralized surface. This observation was supported by alizarin red staining, which revealed staining only along the periphery of the constructs, while the staining in the inner core was not obvious. In contrast, mineralization occurred throughout the COL + CSA constructs, and their cylindrical shape was retained. This was further supported by alizarin red staining observed at both the inner core and the periphery of COL + CSA constructs. Although the quantitative calcium assay (Figure 4B) indicated a higher calcium concentration in the bottom section, except for COL + CSA perfusion, an obvious difference is not distinctly visualized in the qualitative alizarin red‐stained samples.

### 2.5 Gene Expression Profiles of COL and COL+CSA Constructs

Comparisons are made between COL and COL + CSA bone constructs that were cultured in static or in the perfusion bioreactor for up to 60 days. Note that the *Y‐*axis uses a log_10_ scale, and each numerical increase represents a 10‐fold difference. The light‐colored boxes and whiskers represent gene expression in constructs from static cultures, while the dark‐colored boxes represent gene expression in constructs from the perfusion bioreactor (Figure 5). Furthermore, the top (clear) and bottom (lined) sections were analyzed separately to assess the possible effects of scaffold geometry on gene expression. Figure 5 summarizes the gene expression profiles for hMSC (*CD90, Sox2*) and chondrogenic markers (*Sox9, Col2a*), while Figure 6 summarizes the gene expression profiles for hypertrophic chondrocytes (*Cola10a*) and osteoblast markers (*Runx2, ALP, Sp7*). Overall, the potential to direct systematic hMSC differentiation to recapitulate endochondral ossification at the level of gene expression in a perfusion bioreactor was evident. This refers to the transition of chondrogenic differentiation to chondrocyte hypertrophy, followed by osteogenic differentiation. Differences in scaffold geometry have also led to variation in gene expression profiles between static and perfusion cultures, particularly in *Sox2* expression. Furthermore, CSA emerged as a potent inducer of chondrogenic differentiation at 30 days and significantly influenced *Sox2, Col2a, Col10a, Runx2*, and *ALP* gene expression, particularly in perfusion cultures, which showed an earlier onset and elevated gene expression at 30 days.

**FIGURE 5:**
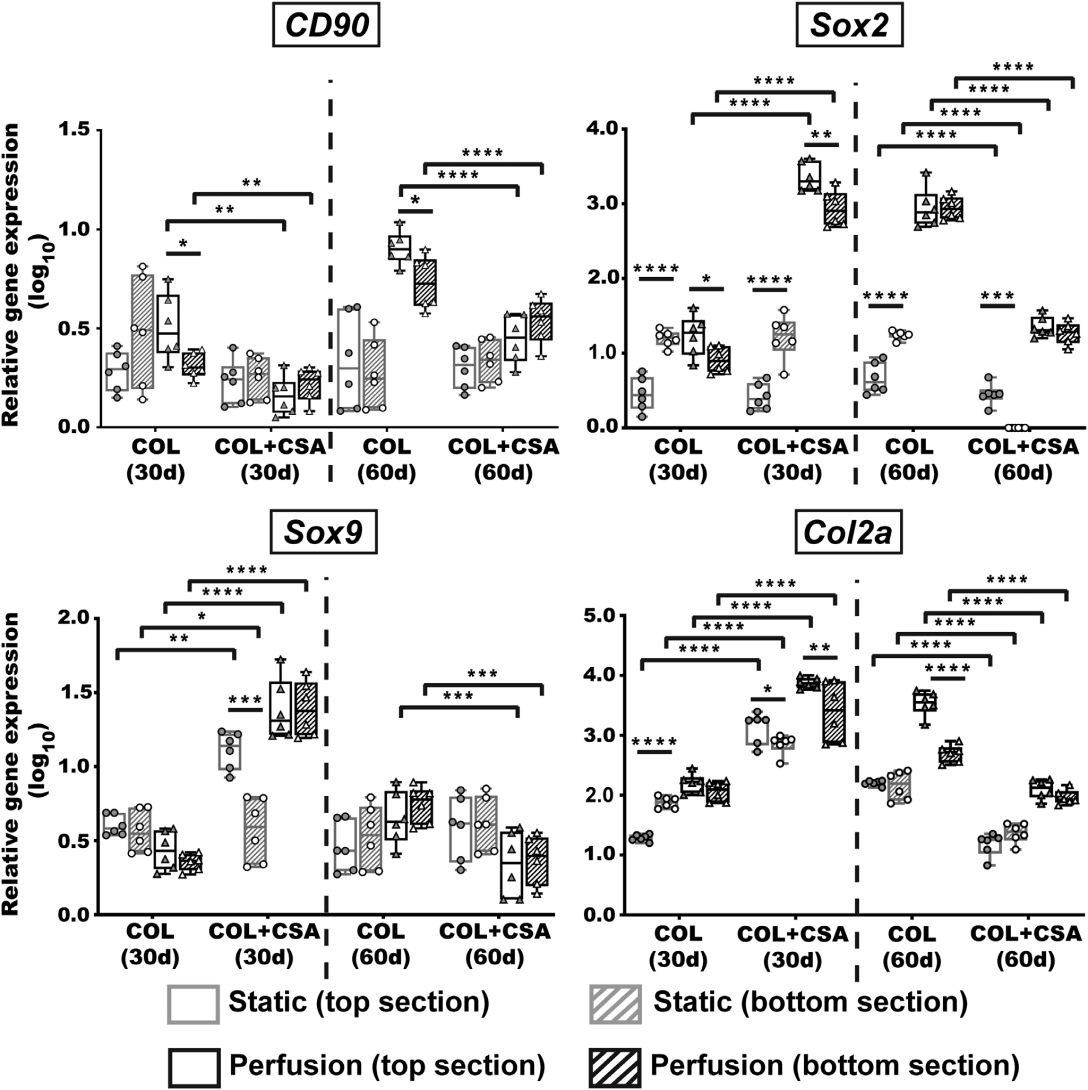
Gene expression profile of the constructs after 30 and 60 days in a perfusion bioreactor and their corresponding static culture. The top cap of each bone construct was dissected, and only the top section (0.19 cm^3^) and the bottom section (0.10 cm^3^) were used for analyses. The gene markers summarized in this figure are for (a) hMSCs: *CD90, Sox2*; and (b) chondrocytes: *Sox9, Col2a*. Two‐way ANOVA and Tukey multiple *t‐*test comparison (*n* = 3, duplicates). The first level of comparison is between the top and bottom sections of the same construct; the second level compares the static and perfusion samples at the same time point; the third level compares the 30 days against the 60 days samples; and the fourth level compares the corresponding COL and COL + CSA samples at the designated time point. * *p* < 0.05, ** *p *< 0.01, *** p < 0.001, **** *p* < 0.0001.

**FIGURE 6:**
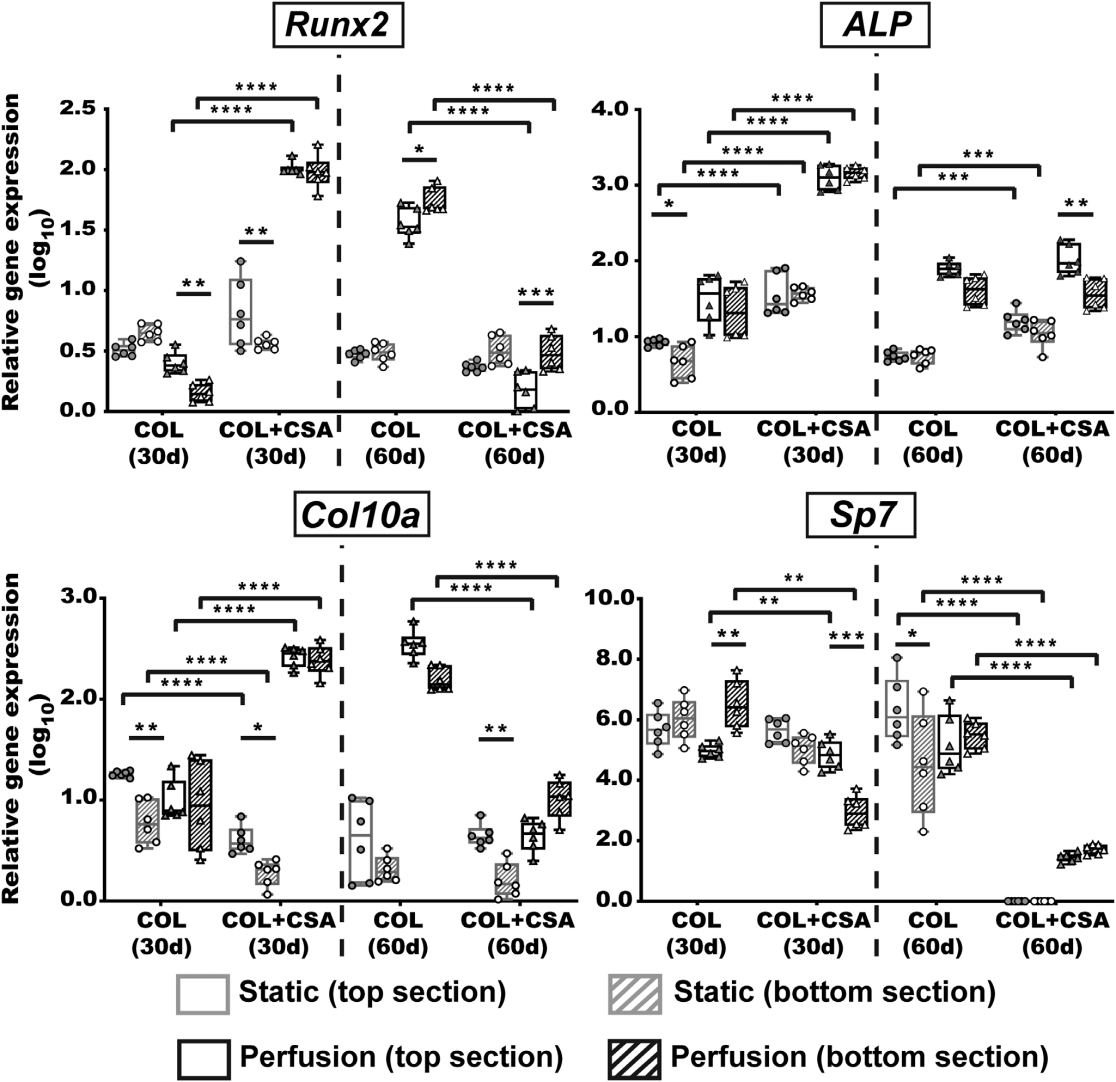
Gene expression profile of the constructs after 30 and 60 days in a perfusion bioreactor and their corresponding static culture. The top cap of each bone construct was dissected, and only the top section (0.19 cm^3^) and the bottom section (0.10 cm^3^) were used for analyses. The gene markers summarized in this figure are for (a) hypertrophic chondrocytes: *Col10a*; (b) osteoprogenitors and osteoblasts: *Runx2, ALP, Sp7*. Two‐way ANOVA and Tukey multiple *t*‐tests (*n* = 3, duplicates). The first level of comparison is between the top and bottom sections of the same construct; the second level compares the static and perfusion samples at the same time point; the third level compares the 30 days against the 60 days samples; and the fourth level compares the corresponding COL and COL + CSA samples at the designated time point. * *p* < 0.05, ** *p* < 0.01, *** *p* < 0.001, **** *p* < 0.0001.

#### 2.5.1 Stem Cell Markers (*CD90* and *Sox2*)

Figure 5 shows that the elevated expression of these stem cell gene markers indicates the continual presence and self‐renewal of hMSCs, which remained positively expressed in all the constructs until 60 days post‐inoculation. Specifically, increased *Sox2* gene expression, a self‐renewal marker, was observed in perfusion cultures. This indicated an active hMSC proliferation mechanism even at 30 days/60 days, and the shear stress from the perfusing medium through the bioreactor had further enhanced it. CSA further accelerated this effect, as elevated *Sox2* expression was already observed in COL + CSA (30 days) perfusion, resulting in gene expression similar to that in COL (60 days) perfusion.

#### 2.5.2 Chondrocyte Markers (*Sox9* and *Col2a*)

Figure 5 shows that both chondrogenic markers were elevated in COL + CSA (30 days) constructs compared to the corresponding COL (30 days) constructs. After 60 days, this effect diminished in the COL + CSA constructs and was evident in both static and perfusion cultures. It is an expected effect, attributed to the removal of CSA supplements from the cell culture medium at 15 days, an attempt to favor osteogenic differentiation until 60 days. Intriguingly, *Col2a* was significantly elevated in COL (60 days) perfusion, and a notable variance was recorded between the top and bottom sections.

#### 2.5.3 Hypertrophic Chondrocyte Marker (*Col10a*)

Significantly elevated expression was documented in the perfusion cultures, and variances due to scaffold geometry (top vs. bottom) were only noted in all static cultures. Similar to the *Col2a* profile, *Col10a* expression was accelerated, and a significant increase was recorded in COL + CSA (30 days) perfusion, highlighting the enhancing effects of CSA supplementation in os medium during the first 15 days. As the effect subsided in COL + CSA (60 days) perfusion, COL (60 days) perfusion had a significant increase in *Col10a* expression. However, this trend was not observed in corresponding static cultures, as seen in the *Col2a* profile. Instead, there were few fluctuations in *Col10a* expression between COL (30 days) static versus COL (60 days) and COL + CSA (30 days) versus COL + CSA (60 days).

#### 2.5.4 Osteoblast Markers (*Runx2*, *ALP*, *Sp7*)

Overall, Figure 6 shows that few fluctuations were recorded in the COL and COL+CSA static cultures after 30 and 60 days. *Runx2* and *ALP* were typically expressed at lower levels in the static cultures than in the corresponding perfusion cultures. Interestingly, the ALP activity assay shown in Figure 4A also corresponded to the *ALP* gene expression profile, with a comparable trend observed across all samples. Further, *Sp7* expression was consistently elevated and comparable to that of the perfusion cultures. An exception to this observation was noted in the COL + CSA bone constructs, which exhibited increased *Sp7* expression in the COL+CSA (30 days) static cultures, but no such increase was recorded in the COL + CSA (60 days) static cultures. Generally, *Runx2* and *ALP* expression were elevated in perfusion cultures and further enhanced by CSA, resulting in increased *Runx2/ALP* expression in the COL + CSA (30 days) constructs. Similar to the chondrocyte and hypertrophic chondrocyte markers, the expression of *Runx2*, *ALP*, and *Sp7* decreased in the COL + CSA (60 days) constructs. In contrast, the COL (30 days) constructs showed lower *Runx2/ALP* expression, with only *Runx2* being substantially elevated in the COL (60 days) constructs.

## 3 Discussion

In this study, multiple complex technologies, including additive manufacturing, biotechnology, and bioreactor‐based cultivation, are serially integrated to generate biomimetic in vitro niches that support engineered bone constructs to acquire properties resembling those of native bone tissue. As a first step, hMSC expansion was successfully scaled up in 250 mL spinner flasks to produce a large quantity of undifferentiated hMSCs in a single passage phase. Next, a systematic approach to generate large collagen‐based constructs with tailored dimensions was developed by integrating 3D printing with cast‐molding and lyophilization (Figure 7).

These hMSCs were then inoculated into specially designed collagen‐based scaffolds at high density and cultivated in a perfusion bioreactor under consistent partial oxygen tension (pO_2_). Additionally, chondroitin sulfate A (CSA) was supplemented to initiate chondrogenic differentiation, whilst a standard osteogenic differentiation medium was used to induce chondrocyte hypertrophy and osteogenic differentiation. With the addition/removal of CSA at the designated time point, this study aimed to achieve a mixture of osteochondral cell lineages from a single source of hMSC, a prerequisite for recapitulating endochondral ossification processes in vitro. Most importantly, exogenous supplementation was limited in this study to facilitate local induction of osteochondral differentiation through cell–cell and cell–matrix interactions, complemented by the dynamic niche within the perfusion bioreactor. Specifically, conventional growth factor supplements were completely excluded; the transition from the chondrogenic to the osteogenic state can be flexibly controlled by CSA supplementation/removal, and osteogenic differentiation was initiated with only 10^−9^ m dexamethasone, rather than the 10^−7^ m used in conventional protocols [34]. Taken together, the working principle and systematic workflow developed in this study also aim to align with the current trend toward sustainability in research, that is, to produce desired end products efficiently while reducing waste/carbon footprint, and the use of animal‐derived products/growth factors [35].

### 3.1 Proficient hMSC Expansion Protocol in 250 mL Spinner Flasks

Cell expansion protocols using small spinner flasks (125 mL) are readily accessible [10, 12, 13, 36]. However, when a substantial quantity of undifferentiated hMSC is desired, cumulative expansion procedures over several hMSC passage phases would be necessary. Considering that cell senescence and reduced multipotency of hMSC are anticipated with increasing passage phases, an optimized working expansion protocol for larger 250 mL spinner flasks was developed based on previous literature [10, 12, 13, 36]. However, the expansion protocol was further refined in this study to achieve high‐quality hMSCs in a single‐passage phase. By employing the inoculation and expansion parameters described in this report, a single 250 mL flask would yield at least 4.4‐fold more hMSC than conventional T175 cell culture flasks, resulting in at least 75 × 10^6^ hMSC available for use (four spinner flasks) after a single passage phase over 12 days. Furthermore, the expansion protocol was successfully applied to hMSC from three different donors, reinforcing its applicability and the efficacy of this approach for generating a large quantity of undifferentiated hMSC from a general source. This is supported by the tabulated cell counts and viability assay results for each donor, as summarized in Figure S2A. These spinner flask‐expanded hMSC also retained the typical spindle‐like cell morphology and demonstrated trilineage multipotency, as they could still undergo adipogenic, chondrogenic, and osteogenic differentiation, just like the hMSC from the static T175 flask, as shown in Figure 1C–E.

In this study, CFD simulations were used to characterize fluid shear in a spinner flask at varying stir rates. A linear correlation was identified between fluid shear and stir rate, and a stir rate of 90 rpm for the rod impeller we used in the 250 mL flask resulted in a maximum shear stress of 7.702 dyn/cm^3^ (Figure 1B). This was close to the optimal conditions identified in previous publications done in a 125 mL flask [12, 13]. The simulation also explained the advantages of rod impellers, which concentrated most of the fluid shear at the ends of the rod and at the glass surface. In contrast to the standard blade impellers with a greater surface area [10], the rod impeller offers homogeneous mixing with fewer high shear regions. Furthermore, the likelihood of hMSC‐laden beads colliding with the impeller and causing cell death was significantly reduced, thereby increasing hMSC expansion efficiency in this setup. With the successful implementation of spinner flask‐derived hMSCs for bone tissue engineering applications here, the current model can be further developed into a digital twin to facilitate the development of the spinner flask for stem cell expansion, achieving greater efficiency and yield. A more sustainable R&D can then be carried out by simply making digital modifications to impeller/flask designs and dimensions, with final in vitro validation on effective simulated designs.

### 3.2 High‐Throughput Fabrication of Tailored Collagen‐Based Scaffolds Through 3D‐Printing and Cast‐Molding

With rapid advancements in 3D printing and bioink development, the direct printing of large bone grafts with tailored dimensions is being extensively studied [37, 38, 39, 40]. Specifically, greater success was achieved with bone cements or inorganic materials that exhibit rapid‐setting properties upon extrusion [37, 38, 39]. For collagen‐based scaffolds or hydrogels that lack initial structural stability, the tendency for printed scaffolds to collapse during printing increases linearly with increasing size, irregular shape, and additional processing [41, 42, 43]. Furthermore, the size of directly printed items is also constrained by the printing axis of the 3D printer [37, 38, 39, 42]. However, the integral approach presented in this report enables the generation of separate molds that can be printed and processed in bulk, thereby overcoming the limitations of producing large, complex collagen‐based scaffolds in large quantities. Due to the limited potential of inorganic materials or cements for cellular infiltration and osseointegration, biological scaffolds remain a more relevant material for bone tissue engineering applications [37, 42, 43]. Hence, an alternative method has been developed to produce large biological scaffolds with tailored dimensions. In this report, a polylactic (PLA) male mold with tailored dimensions was printed and cast in platinum‐cured silicone to derive multiple female molds. Subsequently, rat‐tail type 1 collagen gel was poured into each female mold, lyophilized, and cross‐linked in bulk with 25% glutaraldehyde vapor. As shown in Figures 4 and 6, the collagen scaffolds adopted the tailored shape cast in the female mold and remained structurally stable over time. This approach is more advantageous than direct printing, as it eliminates the need for quick‐setting photo‐crosslinkers and/or supporting materials. Additionally, this method is both cost‐ and time‐effective for rapid prototyping and consistent bulk production of porous biological scaffolds.

### 3.3 Effects of Stable Scaffold Structural Integrity and pO_2_ for Bone Construct Development in the Bioreactor

3D collagen‐based scaffolds with/without CSA, of varying top and bottom dimensions, were seeded with hMSC and cultured within the perfusion bioreactor for 60 days. The key aim is to identify the effects of different combinations on pO_2_ distribution, hMSC proliferation/differentiation, and physical/mechanical properties, as well as their interactions with the niche generated within the perfusion bioreactor. Thereby, laying the path for scaffold designs with more complex geometries and shapes in future studies. Generally, the broad top section and slim bottom section contained comparable absolute collagen (Figure 2A) and CSA content (Figure 2B). This translates to higher collagen concentration per cm^3^ in the bottom section and has a direct impact on the Young´s modulus, where the COL bottom section is mechanically stiffer than the COL top section. Interestingly, this effect was neutralized with CSA supplementation, as the electrostatic interactions of CSA and collagen molecules caused the top and bottom sections of COL + CSA scaffolds to possess similar Young´s modulus [44]. Additionally, the cross‐linked scaffolds demonstrated stable structural integrity, allowing the scaffolds to revert from a clump to their original shape after cell seeding (Figure 7C) and facilitating homogeneous hMSC inoculation in the agarose well. Furthermore, the hMSC‐laden constructs could be easily transferred to the bioreactor using a pair of forceps and oriented within it simply with a glass pipette (Figure 7D).

Despite variability in geometry and volume, the calculated hMSC density per cm^3^ in the top and bottom sections showed only a small difference (Section 2.3.2) that can be correlated with the homogeneous and stable pO_2_ distribution in the perfusion bioreactor (Figure 3B). Generally, a typical hypoxic niche applied in the field had ≥1% oxygen or 7–10 mmHg [45, 46, 47, 48, 49]. As shown in a previous study, the pO_2_ within the agarose bedding that encapsulates the bone constructs remained constant at approximately 127.3 ± 2.9 mmHg (16.7%) after 12 h when a continuous medium perfusion rate of 5.5 mL/min was applied [23]. In this study, pO_2_ in the medium reservoir was also measured, which remained consistent at 137.5 ± 4.9 mmHg (18%) until 60 days. These results demonstrated the competency of perfusion bioreactors to provide a stable oxygen supply. With cellular consumption in mind, the fiber‐optic oxygen sensors were positioned at the midpoint of both the top and bottom sections of a scaffold (Figure S3). It is recognized that these are point‐specific sensors, and the pO_2_ measurements may not be representative throughout the entire scaffold. However, the primary aim of this study is first to monitor significant fluctuations and changes in pO_2_ over 60 days. Therefore, these sensors are preferable and can be easily secured in a fixed position. It is critical to define the pO_2_ within the perfusion bioreactor setup, as a hypoxic niche has been reported to favor chondrogenesis [45, 46, 50], while an oxygenated niche facilitates osteogenesis and mineralization [23, 48]. As reported in our previous study, pO_2_ in this bioreactor can be further manipulated by adjusting the medium perfusion rate, cell density, and perfusion channel design [19, 20]. However, an oxygenated niche was applied (Figure 3B), allowing the enhancing effects of perfusion bioreactor cultivation +/− CSA on osteogenic differentiation, construct mineralization, and the resulting structural properties of the bone construct to be examined. In this study, we established a baseline with 3.5 million hMSCs and perfusion at 5.5 mL/min through four straight 3 mm channels arranged in parallel to the bone constructs and through the agarose bedding [19, 20, 23]. Over the entire course of experimentation in 60 days, pO_2_ was between 58–102 mmHg (Figure 3B). Therefore, hMSCs are generally maintained in an oxygenated niche that is more conducive to osteogenic differentiation.

### 3.4 Comparing Perfusion Bioreactors Against Conventional Static hMSC Culture

In terms of cell numbers over time, all samples have fewer cells than the initial seeding density, especially in the perfusion culture, which decreased by almost 50% at 30 days but slowly increased until 60 days (Figure 3A). This is expected and mainly due to three factors occurring at the early phase: (1) detaching cells due to interstitial fluid shear generated within the bioreactor, (2) release of cytotoxic residual aldehydes through hydrolysis of glutaraldehyde cross‐linked collagen fibrils [51, 52], and (3) the high hMSC number for cell inoculation. Nevertheless, an increase in cell number was observed from 30 to 60 days, and cell viability was confirmed by the LDH assay (Figure S2B).

Based on the appearance of constructs cultivated under static and perfusion conditions (Figure 4D), the enhancing effects of perfusion bioreactor culture on construct mineralization were evident and supported by other literature [53, 54, 55, 56]. This is further supported by quantitative analyses using a calcium assay, which measured the total calcium concentration within the bone construct (Figure 4B), and by the calculated density from µCT images (Table 1). Generally, a similar trend is observed, and both quantitative approaches agree across all samples. As shown in the µCT images, homogeneous mineralization occurred throughout the top and bottom sections of COL (perfusion) and COL + CSA (perfusion) constructs. Although positive alizarin red staining, which indicates calcium accumulation, was observed throughout the COL (static) and COL + CSA (static) constructs, dispersed mineral deposition was noted in their corresponding µCT images. These observations indicate that the accumulated mineral density is lower than that of the hydroxyapatite calibrator implemented for µCT imaging. Unlike the previous reports [53, 54, 55, 56], this study further demonstrated that the structural integrity of constructs was enhanced after 60 days in perfusion cultures. In contrast, severe irregular condensation/shrinkage was observed in the corresponding static cultures (Figure 4D). By encapsulating hMSC‐laden constructs in agarose and facilitating medium perfusion through hollow channels running along these constructs within the agarose (Figure S3), an isolated and stable niche suitable for construct development was achieved in the perfusion bioreactor [19, 20, 23]. Furthermore, direct exposure of the constructs to excessive fluid shear stress at high perfusion rates was mitigated by the agarose, which prevented the initial constructs with weak structural properties from disintegrating. Here, the agarose mimicked connective tissue encapsulating native bone, and the hollow channels corresponded to blood vessels in connective tissue that facilitated mass transfer via convection (liquid flow within the channels) and diffusion (from the channels to cell‐laden scaffolds). Additionally, the perfusion culture minimized construct shrinkage due to hMSC condensation, as demonstrated by static cultures. Such severe shrinkage often limits the generation of large biologically based bone constructs with hMSCs in vitro. Although mesenchymal condensation is a common phenomenon in hMSCs [57, 58, 59], especially at high cell density, it had a lesser impact in this study, since there were comparable absolute cell numbers in both the perfusion and static cultures (Figure 3A). Therefore, this indicates the critical implications of direct osteogenic differentiation versus osteochondral ossification approaches for construct development.

### 3.5 Effects of CSA on Chondrogenic Differentiation and the Structural Integrity of COL + CSA (Perfusion) Bone Constructs

About 4.5% of the initial CSA amount was retained in the COL + CSA scaffolds (Figure 2B). This was sufficient to specify hMSC differentiation and was comparable to previous studies on engineering collagen/glycosaminoglycan (GAG) composites for bone substitutes [26, 60]. Further, COL + CSA scaffolds have significantly higher Young´s moduli than COL scaffolds; an added effect caused by increased electrostatic interactions between the sulfated groups in CSA and the collagen fibrils [44], which benefited the mineralization process [60, 61, 62]. This strengthening effect was further evident in the mechanical stiffness of the COL + CSA top (0.19 cm^3^) and bottom (0.10 cm^3^) sections, especially after cell culture. Despite the higher collagen concentration (µg/cm^3^) in the bottom section, both sections had comparable initial Young´s moduli at 0 days. In contrast, the COL bottom section has a significantly higher Young´s modulus than its top section (Figure 2C). These results indicate that CSA has a stabilizing effect on collagen‐based scaffolds. Unlike most existing literature, which has generated multiphase constructs with uniform dimensions [50, 51, 52], the developed approach reported here yielded large, stable collagen scaffolds with tailored geometries. The longer cell culture duration to 60 days has further elucidated the pronounced effects of geometric design and CSA supplementation on changes in the physical/mechanical properties of bone constructs at later time points (Figure 4). COL + CSA constructs generally supersede the corresponding COL constructs, with the COL + CSA constructs at 60 days remaining cylindrical, more homogeneously mineralized, and exhibiting less shrinkage, especially in the perfusion culture. These are supported by the initial mechanical stiffness (Figure 2C), ALP activity on d30 (Figure 4A), total calcium concentration (Figure 4B), µCT images/calculated density (Table 1), and alizarin red‐stained cross‐sections (Figure 4D).

For COL constructs, the bottom section with a smaller diameter has resulted in a higher Young´s modulus than the top section and has generally accumulated higher calcium concentrations over time (Figure 4B,C). This is coupled with qualitative visualization using alizarin red, which showed inhomogeneous but clustered calcium accumulation within the COL constructs. For the COL perfusion 60 days specifically, alizarin red staining was most pronounced along the periphery of the construct, and µCT images also showed a collapsed structure (Figure 4D). In correlation to the gene expression profiles, earlier onset of *Sp7* gene expression, but reduced *Runx2* at 30 days in COL bone constructs (both static and perfusion) was the most pronounced (Figure 6), while the chondrogenic gene markers, *Sox9* and *Col2a*, also remained low at 30 days (Figure 5). Taken together, these results can be correlated with the processes of intramembranous ossification for compact hard bone formation in vivo, associated with direct osteogenic differentiation via the canonical Wnt/β‐catenin signaling pathway that upregulates *Sp7* independently of *Runx2* [63, 64, 65].

In contrast, the enhancing effect of CSA on chondrogenic differentiation was demonstrated in a previous study [26], and a switch to osteogenic differentiation at the genetic level can be achieved simply by removing CSA. Similarly, this study established this and further showed that, at the level of gene expression, the transition proceeded from chondrogenic differentiation (*Sox9, Col2a*) to an intermediate state with chondrocyte hypertrophy (*Col10a*), followed by osteogenic differentiation (*Runx2, ALP*). This was most evident in the perfusion culture and can be correlated with the processes of endochondral ossification involved in long bone formation. Through the cyclic occurrence of endochondral ossification and osteoclast‐mediated bone remodeling, long bones with greater volume and distinct structural properties are formed [66, 67, 68]. Briefly, the initial effects of CSA may be explained by its capacity to bind and stabilize growth factors such as TGF‐β1 and various BMPs produced by cells within the extracellular matrix, thereby directing chondrogenic maturation and subsequent chondrocyte hypertrophy [69, 70]. The intermediate state of chondrocyte hypertrophy is also marked by the gene expression of *Col10a*, which is commonly used *in vitro/in vivo* [71, 72, 73] and is indispensable for the subsequent transition to osteogenic differentiation [74, 75]. As evident in this study, the physical/mechanical properties and mineralization of bone constructs at 60 days are significantly affected by whether chondrogenic differentiation occurs before or after the osteogenic differentiation step. Thereby underscoring the importance of the initial chondrogenic differentiation process for successful derivation of bone substitutes meant for long bone replacement/regeneration.

In summary, these results highlight the critical cellular factors required to establish biomimetic niches in vitro for deriving bone‐like constructs. In addition to direct incorporation of multiple osteochondral cell lineages in large quantities within a bone construct, the sequence and duration of each cell population's presence in the construct are equally important. Therefore, more precise variations to the existing cultivation parameters could be implemented to further stipulate the differentiation processes. For example, acidic chondrogenic differentiation medium [76, 77] and hypoxia [78] achieved through a low perfusion rate can be applied in conjunction with CSA to further enhance chondrogenic differentiation at the initial stage. Subsequently, the perfusion rate can be increased to create an oxygenated niche, and a slightly alkaline, pH 7.6, osteogenic differentiation medium can be introduced to favor chondrocyte hypertrophy and osteogenesis [77]. Alternatively, exogenous electric field stimulation could also be implemented to enhance and specify osteogenic differentiation [15, 79].

## 4 Conclusions

The feasibility of supporting near‐physiological in vitro niches by integrating multiple technologies to generate large, tailored bone constructs from collagen‐based scaffolds was demonstrated in this study. Furthermore, the combination of applied biochemical and physical stimuli was sufficient to induce osteochondral differentiation in hMSCs without explicit exogenous growth factor supplementation. Depending on the scaffold used (COL/COL + CSA), the processes of intramembranous and endochondral ossification can be recapitulated in vitro, at the level of gene expression, from a single source of hMSC. Furthermore, long‐term culture in the perfusion bioreactor supports the continual development of bone constructs, ultimately yielding mineralized bone constructs with preserved structural integrity. Based on these results, additional variability in medium pH and pO_2_, the design and composition of composite scaffolds, and co‐culture with endothelial cells could be incorporated into the existing approach to achieve bone constructs that more closely recapitulate native bone tissue. Most importantly, the distribution and presence of different cell types should also be validated at the level of protein expression/immunohistology and compared with native bone tissue as a next step.

## 5 Materials and Methods

### 5.1 Bone Marrow‐Derived Human Mesenchymal Stromal Cells (hMSC)

Bone marrow aspirates were isolated from healthy male donors (20–40 years old), and the hMSCs were isolated by Prof. Dr. Bornhäuser from the University Hospital Carl Gustav Carus Dresden. The procedure was completed in compliance with Ethics approval #EK263122004, and Ethics approval #EK367072019 granted access and outlined the guidelines for the handling/use of the hMSC in this study. Both ethics approvals were assessed and granted by the Ethics Commission of Dresden University of Technology to Prof. Bornhäuser and Dr. Lee respectively. The hMSC from three donors were used in the experiments. Briefly, the hMSCs were positively selected for CD105, CD73, CD166, CD90, and CD44 markers and were negative for hematopoietic stem cell markers CD34 and CD133 by flow cytometry [80]. The hMSCs were maintained at 37°C, 5% CO_2_ in the expansion (exp) medium containing Dulbecco's modified Eagle's medium (DMEM) low glucose, 10% fetal calf serum (Capricorn Scientific), 2 mm l‐glutamine, and 100 I.U./mL penicillin‐streptomycin (all from Sigma‐Aldrich, unless otherwise specified).

### 5.2 Preparing CultiSpher‐S Beads and Scaled‐up hMSC Expansion in Spinner Flask

CultiSpher‐S beads (Sigma‐Aldrich) were used to increase the potential surface area for cell attachment and to scale up the expansion of anchorage‐dependent hMSCs. Pre‐conditioning of 250 mL spinner flasks (NDS Technologies) with silicone (Sigmacote), bead preparation, cell inoculation, and cell culture conditions were performed as previously described [12, 13]. A rod impeller (Ø: 15 mm, length: 65 mm) was used to achieve a similar calculated fluid shear stress. Additionally, the fluid dynamics in this modified setup were mathematically modeled. Briefly, 0.3 g dry CultiSpher‐S beads were hydrated in a silicone‐coated Pyrex flask with 100 mL Dulbecco's phosphate‐buffered saline (DPBS) at room temperature (RT) for 3 h; followed by autoclaving. The beads were isolated using cell strainers (70 µm pore size; Falcon) and washed twice by passing 15 mL of warm, serum‐free DMEM (37°C) through the cell strainer. They were then transferred to a 250 mL spinner flask. The beads were equilibrated overnight with stirring at 90 rpm in 90 mL of serum‐free inoculation medium (DMEM; pH 7.6–7.65, 2 mm l‐glutamine, 100 I.U./mL penicillin‐streptomycin) at 37°C and 5% CO_2_. The next day, 2 × 10^6^ hMSC were inoculated into each spinner flask to achieve a calculated bead: cells ratio of 1: 10. An intermittent stirring regime (3 min stirring at 90 rpm, 27 min pause) was applied for 6 h. Upon completion, 10 mL of FBS was added, and the volume was topped up to 250 mL with warm exp medium. Cell expansion was carried out with continuous stirring at 90 rpm, 37°C, 5% CO_2_ for 12 days. Medium change was performed after 6 days by aspirating 200 mL of medium after the CultiSpher‐S settled to the bottom and replaced with an equal volume of fresh exp medium.

To harvest the hMSC, the beads were split between two cell strainers, followed by a washing step with 30 mL warm DPBS (37°C). The beads from two cell strainers were pooled and collected in a fresh 50 mL conical tube by passing 20 mL 0.05% trypsin‐EDTA (Sigma‐Aldrich) through the reverse side of each cell strainer. The hMSC‐laden beads were incubated in the water bath at 37°C for 10 min, with periodic mixing by inverting every 2 min. Subsequently, the suspension was passed through a cell strainer (70 µm pore size) to separate the detached hMSCs from the beads, and the trypsin‐EDTA‐hMSC suspension was deactivated using an equal volume of exp medium. Upon isolation, total cell numbers were counted using an automated cell counter (EVE, VWR). Trypan blue was mixed with the cell suspension at a 1:1 ratio to attain viable cell counts. Cell viability was further assessed using the lactate dehydrogenase (LDH) assay described in Section 5.6. The cell suspensions were applied for subsequent hMSC characterization steps. Next, static culture with the same batch of hMSC was set up in two T175 flasks (1 × 10^6^ cells per flask) for comparison. The hMSCs from T175 and the spinner flask were isolated after 12 days and seeded in 6‐well plates. The total cell numbers, cell morphologies, and the expression of hMSC gene markers—*CD90* and *Sox2*—were assessed. Furthermore, standard adipogenic/chondrogenic/osteogenic trilineage differentiation assays were applied to evaluate hMSC multipotency [81, 82], which was visualized using oil red O, alcian blue, and alizarin red staining.

### 5.3 Mathematical Model of Fluid Dynamics in a Spinner Flask

The hydrodynamic environment within a 250 mL spinner flask was characterized using CFD simulations performed in ANSYS CFX 2021 R1 (ANSYS Inc., Canonsburg, PA). The flask, filled with 250 mL of cell culture medium, had an inner diameter of 80 mm. A cylindrical impeller (15 mm diameter, 65 mm length), positioned centrally, induced mixing. The fluid was modeled as a Newtonian, incompressible liquid with the thermophysical properties of culture medium at 37°C: density *ρ *= 1006 kg/m^3^, kinematic viscosity *ν* = 8.45 m^2^/s, approximating those of DMEM‐based formulations. Further specifications of the spinner flask and parameters applied in this study are summarized in Figure S4.

A single‐phase flow model was adopted, assuming that cells and microcarriers had a negligible influence on the bulk fluid dynamics. The turbulence model employed was the scale‐adaptive simulation shear stress transport model, designed to capture unsteady flow structures [83]. To accurately capture impeller motion, the transient rotor‐stator approach was used to couple the rotating impeller region with the stationary flask domain. The computational mesh consisted of 8.85 × 10^6^ elements, with local refinement near the impeller. Boundary conditions included no‐slip on all solid walls, a rotating wall on the impeller, and symmetry at the fluid–air interface. The liquid surface was treated as non‐deformable based on experimental observations showing minimal free‐surface deflection at the tested agitation speeds. The initial velocity in the domain was set to zero.

Transient simulations were performed at three impeller speeds: 60, 90, and 120 rpm. The time step was adjusted based on rotational speed to maintain temporal resolution corresponding to an angular displacement of 2° per step during the initial transient phase. After achieving approximate periodic flow behavior, the time step was refined to 1° per step to capture fine‐scale unsteady flow features. This resulted in time steps of 2.91 × 10^−4^, 1.93 × 10^−4^, and 1.45 × 10^−4^ s for 60, 90, and 120 rpm, respectively. The total simulation time covered 40 impeller rotations, with flow statistics collected during the final 10 rotations for analysis. The pressure–velocity coupling was solved using a fully coupled algorithm, while momentum equations were discretized using a second‐order upwind scheme. Turbulence transport equations were solved with a bounded second‐order approach. From the resulting steady flow fields, key hydrodynamic parameters—such as volume‐averaged shear rate and maximum shear stress—were computed to characterize the fluid environment experienced by cells and microcarriers.

### 5.4 Standardized 3D Printed Molds for Sustainable Fabrication of Tailored Collagen Scaffolds

The 3D model of a triple cylindrical male mold (Cap: Ø 10 mm, height (*ht*) = 20 mm; Top: Ø 8 mm, ht = 15 mm; Bottom: *Ø* 5 mm, ht = 25 mm) was constructed using SolidWorks 2013 and exported as STL files. The printing was performed on a Zortrax M300 Dual & HEPA, using red polylactic acid (PLA) filament at an extrusion temperature of 210°C on a 60°C heated build platform, with a layer height of 0.1 mm and a 50% fill density (Figure 7A; left). Next, the printed PLA molds were cast in platinum‐cured silicone (Silikonfabrik, Germany) and left to cure in an oven at 60°C for 3 h (Figure 7A; right). The PLA mold was subsequently removed, and the silicon mold was cleaned in an ultrasonic bath for 15 min before being used to fabricate the type 1 collagen (COL) scaffolds with tailored dimensions in bulk. Briefly, each silicone mold was designed to hold 2 mL of collagen mix (1 mg/mL). Two variants, COL and COL + Chondroitin sulfate A (CSA), were prepared. For COL + CSA scaffold, 2 mg/mL rat‐tail type I collagen (COLI, Corning) was prepared in cold (4°C) 10 mm acetic acid and mixed with equal volume of pre‐warmed (37°C) fibrillogenesis buffer (50.15 mm Na_2_HPO_4_ + 11.17 mm KH_2_PO_4_, pH 7.4), containing 2 mg/mL CSA (bovine trachea derived, Sigma Aldrich) by vortexing. The COL scaffold was prepared similarly, except that CSA was omitted from the fibrillogenesis buffer. These were incubated at 37°C for 18 h, followed by lyophilization in a Christ Epsilon 2‐4 LSC freeze dryer for 19 h (Figure 7B). The lyophilized scaffolds were removed from the silicone molds and cross‐linked with 25% glutaraldehyde vapor in an air‐tight Tupperware at 40°C for 48 h. Next, the scaffolds were ventilated in a biochemical fume hood at RT for at least 3 days to remove residual glutaraldehyde and equilibrated in 50 mL serum‐free DMEM (Gibco) at 37°C for 24 h; prior to the cell culture experiments.

**FIGURE 7:**
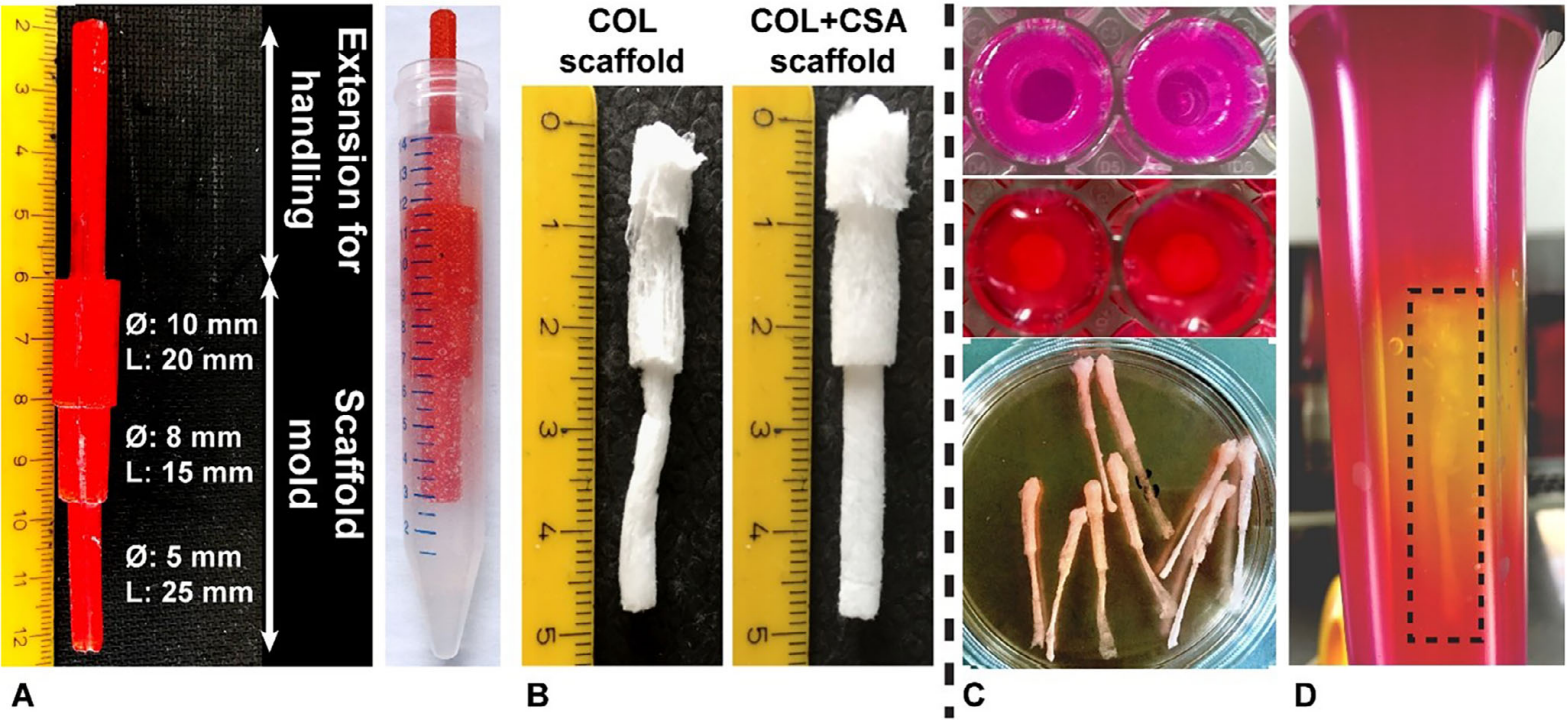
Preparing large, tailored collagen‐based scaffolds through 3D‐printing and cast‐molding. (A) A polylactic acid (PLA) male mold of tailored dimensions was 3D‐printed and cast in platinum‐cured silicone to generate batches of consistent female molds. (B) Lyophilized collagen scaffolds (COL) and chondroitin sulfate A (CSA)‐embedded scaffolds (COL + CSA) with tailored dimensions. (C) Top: Agarose wells (*Ø* = 6 mm, depth 12 mm) for hMSC inoculation. Middle: Equilibrated scaffolds with 3.5 × 10^6^ hMSC/250 µL exp medium in an agarose well. Bottom: hMSC‐laden constructs that returned to their original shape within minutes after being transferred to a Petri dish with warm exp medium. (D) hMSC‐laden constructs encapsulated within the agarose bedding in the perfusion bioreactor.

### 5.5 Determining Collagen, CSA/Calcium Content and the Young´s Modulus of Scaffolds

The equilibrated scaffolds were used, with the top and bottom sections of COL/COL + CSA scaffolds assessed separately. The collagen content was determined by Fluoraldehyde o‐Phthaldialdehyde reagent solution assay (OPA, ThermoFisher). Each specimen was submerged in 900 µL TES‐buffer (50 mm TES + 0.36 mm CaCl, pH 7.4), and incubated at 100°C for 10 min, and cooled at RT for 15 min. Next, 100 µL 1% (w/v) collagenase A/TES solution was added, and collagen digestion was initiated by incubating the specimens on a shaker at 37°C for 18–24 h. Subsequently, the substrates were vortexed, and 20 µL was aliquoted into a 96‐well microplate, followed by 200 µL OPA solution per well. The fluorescence intensity was measured with excitation wavelength: 340 nm and emission wavelength: 440 nm. The raw values were tabulated against a standard curve.

Dimethyl‐methylene blue (DMMB) assay was used to quantify CSA content in COL + CSA scaffolds [84]. First, each specimen was submerged in 400 µL 1% (w/v) Papain/DPBS solution and incubated at 60°C for 24 h. The substrate was briefly vortexed, and 40 µL was aliquoted in a transparent 96‐well microplate, followed by 250 µL DMMB solution (2.1 mg DMMB in 100 mL 0.002% (w/v) sodium formate/DPBS, pH 1.5) in each well. Colorimetric measurements were performed at 595 nm, and the raw values were tabulated against a standard curve. Further, the values from COL + CSA were normalized against COL scaffolds.

The samples retrieved after 60 days in respective conditions were washed thrice in 2 mL PBS (no Ca^2+^ and Mg^2+^) to remove residual Ca^2+^ from the cell culture medium. Subsequently, 0.5 mL 0.25 m HCl prepared in PBS (no Ca^2+^ and Mg^2+^) was added to each sample in a 1.7 mL Eppendorf tube and kept in the dark at 4°C for 48 h to dissolve the calcium deposits. The complexometric method with Fluitest CA CPC (Analyticon) was used, and the substrate solution and standards were prepared according to the manufacturer's protocol. Briefly, the supernatant (10 µL) from the respective sample was mixed with 300 µL of substrate solution in a 96‐well plate. The reaction was left in the dark at RT for 10 min, and the absorbance was measured at 570 nm on a microplate reader.

To determine Young´s modulus, the equilibrated scaffolds were cut with a scalpel to obtain cylindrical samples with a height: diameter ratio of 2:1. The mechanical stiffness of each specimen was measured on the CellScale MicroTester (Waterloo, Canada). To derive a stress–strain plot, each specimen was compressed for three cyclic periods of 50% magnitude in serum‐free DMEM at 37°C. The Young´s modulus was calculated from the mean gradient of the initial linear region of the stress–strain plot and tabulated as a bar chart.

### 5.6 Deriving hMSC‐Laden Constructs and Setting up the Perfusion Bioreactor and Colorimetric Analytics

After equilibrating the scaffolds in serum‐free DMEM at 37°C, 5% CO_2_ for 24 h, the entire scaffold was confined within a cylindrical agarose well (*Ø*6 mm, depth: 12 mm; Figure 7C) and 3.5 × 10^6^ hMSC suspended in 250 µL exp medium were added drop by drop into each agarose well. The scaffolds were incubated at 37°C, 5% CO_2_ for 6 h and periodically mixed every 30 min with a pipette tip to obtain hMSC‐laden constructs for bioreactor transfer. The perfusion bioreactors were prepared as described in previous literature [19]. Briefly, two cultivation platforms with the same design and height of the 3D‐printed PLA mold, but with a 50% reduction in diameter, were cast in the 1.5% w/v agarose/DMEM bedding within each perfusion bioreactor. This was done specifically to accommodate the final form of the scaffolds after shrinkage during cross‐linking in 25% glutaraldehyde vapor and swelling post‐equilibration in serum‐free DMEM. To set up the bioreactor culture, hMSC‐laden constructs were transferred from the agarose wells to a 100 mm petri dish with warm exp medium for 1 min (Figure 7C). These constructs were handled with a pair of forceps and transferred into individual precast cultivation platforms containing 750 µL of type 1 COL solution (1 mg/mL in fibrillogenesis buffer), resulting in a change in the phenol red color to yellow in the agarose bedding (Figure 7D). This was followed by adding cooled agarose (∼42°C) on top to complete the encapsulation procedure. Although the difference in density of the agarose‐COL phases would keep them separated as the agarose solidifies, the topmost 5 mm Cap on the construct acted as a buffer and was therefore cut off and excluded from most quantitative analyses.

The osteogenic differentiation (os) medium was composed of exp medium supplemented with 10 mm β‐glycerophosphate, 50 µg/mL l‐ascorbic acid, and 10^−9^ m dexamethasone. For bioreactors containing COL + CSA scaffolds, the os medium was further supplemented with 1 mg/mL CSA to reinforce chondrogenic differentiation, but only for the first 15 days. Between 16–60 days, both setups were maintained only in os medium. This was done to demonstrate the potency of CSA in inducing chondrogenic differentiation and the importance of recapitulating endochondral ossification processes for bone‐substitute development. Each bioreactor was perfused with 170 mL os medium held in a modified T75 flask medium reservoir at 5.5 mL/min, and medium changes were performed every 15 days. Corresponding static cultures were maintained in T75 cell culture flasks containing the same differentiation medium (170 mL) and incubated at 37°C and 5% CO_2_. Samples were collected after 30 and 60 days.

Cell number (DNA): Cell seeding proficiency on the construct was validated 24 h post‐inoculation using the Quant‐iT PicoGreen assay. Briefly, samples were retrieved from the bioreactor and T75 flask. The cap (topmost 5 mm) was omitted, and the top and bottom half‐sections of an hMSC‐laden construct were placed in separate Eppendorf tubes with 1 mL lysis buffer (1% v/v TritonX‐100/DPBS) on ice for 30 min, followed by a quick vortex. The double‐stranded deoxyribonucleic acid (dsDNA) was quantified by adding 190 µL Pico‐green working solution (1: 380, Pico‐green: TRIS‐EDTA buffer) to 10 µL cell lysate in a 96‐well plate and incubated in the dark at RT for 5 min. Fluorescence intensity measurements were performed on a TECAN microplate reader with an excitation wavelength of 485 nm and an emission wavelength of 535 nm, and the raw values were tabulated against a standard curve. Two constructs were retrieved from 3 different sets of experiments, each with a different hMSC donor (*n* = 6), and measurements were performed in triplicate. Similarly, samples were collected after 30 and 60 days to measure the cell numbers in the respective samples.

Cell viability (LDH): Lactate dehydrogenase (LDH) activity was determined using the LDH cytotoxicity detection kit (Takara, Saint‐Germain‐en‐Laye, France). An aliquot of the cell lysate prepared above for DNA measurements was further diluted 1:50 in lysis buffer to limit over‐saturation and mixed with LDH substrate buffer. The enzymatic reaction was stopped with 0.5 m HCl after 15 min in the dark at RT. Likewise, the calibration curve was prepared using cell lysates of defined cell numbers, and an additional 1:50 dilution was also applied to cell numbers higher than 100 000 cells. For analysis, absorbance was measured at 492 nm on a Tecan microplate reader. The results for the cell viability assay are included as Figure S2.

Alkaline phosphatase (ALP) activity for osteogenic differentiation: ALP activity was determined using 4‐nitrophenyl phosphate (Sigma‐Aldrich) as the substrate and the manufacturer´s protocol. Briefly, 100 µL of a 1 mg/mL substrate solution (1 mm 4‐nitrophenylphosphate in 0.1 m diethanolamine, 0.1 % Triton X‐100, and 1 mm MgCl2, pH 9.8) was added to 25 µL of cell lysate that was also prepared for DNA/LDH analyses. After 60 min of incubation at 37°C on a shaker, the reaction was stopped with 50 µL of 0.5 m NaOH. The absorbance was measured at 405 nm on a Tecan microplate reader, and the calibration curve was generated from a defined range of 4‐nitrophenyl (PNP) concentrations, also incubated at 37°C for 60 min. The calculated values were normalized to the respective cell number (DNA) for each sample for tabulation.

### 5.7 Customized Data Acquisition (DAQ) Setup for Oxygen Tension (pO_2_) Recording

Partial oxygen tension (pO_2_) and temperature within the perfusion bioreactor and the medium reservoir were measured using a multi‐channel OxylitePro (Oxford Optronix) to facilitate concurrent measurements at multiple positions in real time. Furthermore, each fiber‐optic probe has both pO_2_ and temperature sensors, enabling parallel recording. An intravenous (IV) catheter with a 20G needle (Vasofix; B Braun) was used to poke through the bioreactor body, and the tip was positioned at the mid‐point of the top/bottom section of a construct, as shown in Figure S5A. As for the medium reservoir, the IV catheter was inserted through the tubing for the outgoing medium, which perfused into the bioreactor.

To enable efficient data collection, a customized DAQ setup was constructed using an Arduino and a PLX‐DAQ Excel spreadsheet. The setup includes an Arduino UNO (ATmega 328P), a high‐performance Atmel microcontroller based on an open‐source platform that employs the C^++^ programming language. The flow process for this setup is illustrated in Figure S5B. The pO_2_ and temperature can be recorded continuously on a PC via an analogue output port on the OxylitePro, which features a 15‐pin female D‐type connector. A dedicated data cable (NX‐BNC) was utilized to acquire data through an analogue read from the Arduino UNO. The connections of the Arduino pins to the BNC connectors are summarized in a table (Figure S5B). The microcontroller unit automates switching the OxylitePro on and off via an electromagnetic switch (relay). The pO_2_ and temperature were measured at 15‐second intervals over 5 min, and the values recorded by the respective sensors were logged in an Excel file. pO_2_ and temperature were monitored every 4 h during the first 24 h (1 day). At subsequent time points (3, 30, and 60 days), recordings were taken hourly over a 4 h period, and the mean values were plotted as a line graph to illustrate oxygen transfer at a perfusion rate of 5.5 mL/min in the bioreactor throughout the cell culture period.

### 5.8 Cryosectioning and Histological Staining

At designated time points, the bone constructs were removed from the bioreactor/T175 flask and fixed in 4% (v/v) formaldehyde/DPBS at 4°C overnight. Subsequently, the top and bottom sections were separated and washed twice with cold DPBS. Next, the constructs were infiltrated with optimum cutting temperature compound (O.C.T.) medium at RT for 20 mins. Individual sections were embedded in Cryomold with O.C.T., frozen rapidly in 2‐methylbutane on dry ice, and stored at −80°C. The frozen blocks were sectioned at −25°C using a cryostat, and 30 discontinuous cross‐section slices (12 µm) were collected from random positions within the construct onto Superfrost Plus microscope slides. This was followed by air‐drying at RT for 10 mins. The glass slides with cryosections were stored at −80°C until further processing. For histological staining, the frozen slides were air‐dried at RT for 10 min and dipped in distilled water (dH_2_O) for 3 min to dissolve the O.C.T. One bone construct was retrieved for each hMSC donor to complete this analysis (*n* = 3), and the representative images are shown in Figure 4.

Alizarin red staining was used to visualize calcium accumulation, an indicator of osteogenic differentiation and construct mineralization. Therefore, the sections were submerged in 2% w/v Alizarin red S solution (Sigma‐Aldrich), pH 4.1 at RT for 3 min, and dipped in acetone 20 times to remove excess stain. This was followed by a dehydration step in a series of alcohol washes (70% v/v, 90% v/v, 100% v/v ethanol/dH_2_O) and in Histochoice clearing solution (Sigma Aldrich) each at RT for 3 mín. Next, 20 µL Permount solution (Fisher Scientific) was added to each glass slide, and a glass coverslip was mounted on top. The glass slides were air‐dried at RT overnight, followed by light microscopy (AxioVert, Zeiss).

### 5.9 Micro‐Computed Tomography (µCT)

To analyze the overall degree of mineralization, the constructs (60 days) were scanned using micro‐computed tomography (vivaCT 75, SCANCO Medical, Brüttisellen, Switzerland) with an isotropic voxel size of 20 µm (45 keV, 88 µA, 130 ms, 1000 projections). This was performed prior to the histological studies to enable accurate comparisons. One sample was retrieved from each donor for analysis, and the representative images are shown in Figure 4D. The scanner was calibrated weekly using hydroxyapatite phantoms. For analysis, image programming language (IPL) software from SCANCO Medical was used. Contours were drawn manually around the sample surface to determine the total volume (TV) of each sample. The threshold for mineral volume content (MV) of the samples was set to 10 mg HA/cm^3^, and MV/TV was calculated to define the mineral volume ratio. This value was used to estimate the amount of hydroxyapatite present in each construct.

### 5.10 Total RNA Isolation, cDNA Synthesis and Quantitative Polymerase Chain Reaction (qPCR)

Bone constructs were retrieved at 30 and 60 days from three independent experiments using hMSCs from three donors. The top and bottom section of each bone construct was separated and homogenized in 1 mL TRIzol (Invitrogen) at RT for 5 min. Generally, 1500 µg total RNA was used to synthesize cDNA in a 50 µL reaction solution of AffinityScript qPCR cDNA synthesis kit (Agilent Technologies). At the end, an additional 100 µL DNAse/RNAse‐free water was added to achieve a final cDNA concentration of 10 µg/mL. All procedures were performed according to the manufacturer's protocol. A series of osteochondral markers was investigated, and the primer sequences were designed using the Primer3 program [85]. The gene sequences are tabulated in Table 2 and include: (A) stem cells: *CD90*, *Sox2*, (B) chondrocytes: *Sox9, Col2a*, (C) hypertrophic chondrocytes: *Col10a*, (D) osteoprogenitors/osteoblasts: *Runx2, ALP, Sp7*, (E) endogenous control: *36B4, β‐actin*. The samples were analyzed using Brilliant II SYBR green qPCR Mastermix with low ROX (Agilent Technologies) in triplicate on the Agilent Stratagene Mx3005P cycler. Relative gene expression against undifferentiated hMSCs was calculated using the 2^‐∆∆CT^ method [86]. The mean CT values from both endogenous controls were used to evaluate gene expression.

**TABLE 2:** Primer sequences for qPCR analysis.

Lineage	Gene	Primer sequence 5´‐ 3´ forward (F), reverse (R)	size (bp)	Annealing temp (°C)
**MSC**	*CD90*	F—agagctgcttctgtctggtt R—gctctcactctccatcaggt	101	58
**Progenitors**	*Sox2*	F—aagtactggcgaaccatctc R—attaccaacggtgtcaacct	121	56
**Chondrocyte**	*Sox9*	F—gaaagagaggaccaaccaga R—cttggaaatttgggtacgag	134	56
**Mature chondrocyte**	*Col2a*	F—ggatgggcagaggtataatg R—acagattatgtcgtcgcaga	100	56
**Hypertrophic chondrocyte**	*Col10a*	F—attatgacccaaggactgga R—caggggtgccattcttatac	120	56
**Osteoblast**	*Runx2*	F—acccaagcagaatttagcag R—cctacaaaggtgggtttgag	85	56
**Osteoblast**	*ALP*	F—acagacaagaagcccttcac R—tggagacattctctcgttca	85	56
**Osteoblast**	Osterix (*Sp7)*	F—cctacccatctgactttgct R—tctagctgcccactatttcc	133	57
**Endogenous**	*36B4*	F—acggacaggattgacagatt R—gccagagtctcgttcgttat	118	56
**Endogenous**	*β‐Actin*	F—agcaggagtatgacgagtcc R—gttttctgcgcaagttaggt	114	59

### 5.11 Statistical Analysis

The values represented mean ± SD. Student *t‐*test, two‐way ANOVA, and Sidak´s multiple *t*‐test comparison were used to evaluate the variance, and the types of statistical test used for respective analyses are mentioned in the figure legends of the respective diagrams with *α* = 0.05. Generally, technical triplicates were performed for DNA, LDH, calcium, and qPCR analyses, but only the mean values were used for statistical analyses. For Figures 3 and 4, different symbols are used to represent the different types of statistical analyses performed, namely: (#) compares the corresponding sections of COL and COL + CSA at the designated time point, (*) compares the top and bottom sections of the same construct or between the top and bottom sections of COL and COL + CSA.

## Author Contributions

Conceptualization, P.S.L., F.B., H.J., R.A., and B.K. Methodology, A.P., S.R., M.K., S.M.I., P.N.Y., and R.B. Validation, P.S.L., F.B., H.J., R.A., and B.K. Formal analysis, A.P., S.R., M.K., S.M.I., P.N.Y., and R.B. Investigation, A.P., S.R., M.K., S.M.I., P.N.Y., and R.B. Resources, P.S.L., H.J., and B.K. Writing – original draft, F.B., A.P., S.M.I., and R.B. Writing – review & editing, P.S.L., S.R., H.J., R.A., and B.K. Supervision, P.S.L., H.J., R.A., and B.K. Project administration, P.S.L., H.J., and B.K. Funding acquisition, P.S.L., H.J., and B.K.

## Funding

Deutsche Forschungsgemeinschaft (DFG, German Research Foundation) Project—#460388836, DFG SFB 1270/1,2 – 299150580 ELAINE, European Regional Development Fund—Project Excellence in Regenerative Medicine (No. CZ.02.01.01/00/22_008/0004562), Internal Grant Agency of Jan Evangelista Purkyně University in Ústí nad Labem (project no. UJEP‐SGS‐2023‐53‐005‐3, project no. UJEP‐IGA‐2024‐53‐003‐2).

## Conflicts of Interest

The authors declare no conflicts of interest.

## Supporting information




**Supporting File**: advs76590‐sup‐0001‐SuppMat.pdf.

## Data Availability

The experimental data required to reproduce these findings will be available upon request through email at the discretion of the corresponding authors.
