# Supplementary material for: Sustainable Fabrication of Tailored Bone Substitutes: From High‐Throughput Scaffold Manufacturing, Scaled‐Up HMSC Expansion to Dynamic Cultivation in a Perfusion Bioreactor
Source: Adv Sci (Weinh). 2026 Jul 23:e23846. Online ahead of print. doi: 10.1002/advs.202523846 (PMC13395396; doi:10.1002/advs.202523846)
Supplement: Supplementary file 1 — Supporting File: advs76590‐sup‐0001‐SuppMat.pdf. [file ADVS-9999-e23846-s001.pdf]

Supporting Information

Title: Sustainable fabrication of tailored bone substitutes: From high-throughput scaffold manufacturing, scaled-up hMSC expansion to dynamic cultivation in perfusion bioreactor

Franziska Alt, Anna Paříková, Sandra Rother, Martin Kantor, Salman Muhammad Ilyas, Ricardo Bernhardt, Pavel Ndjawa Yomi, Havlica Jaromir, Revathi Appali, Benjamin Kruppke, Poh Soo Lee

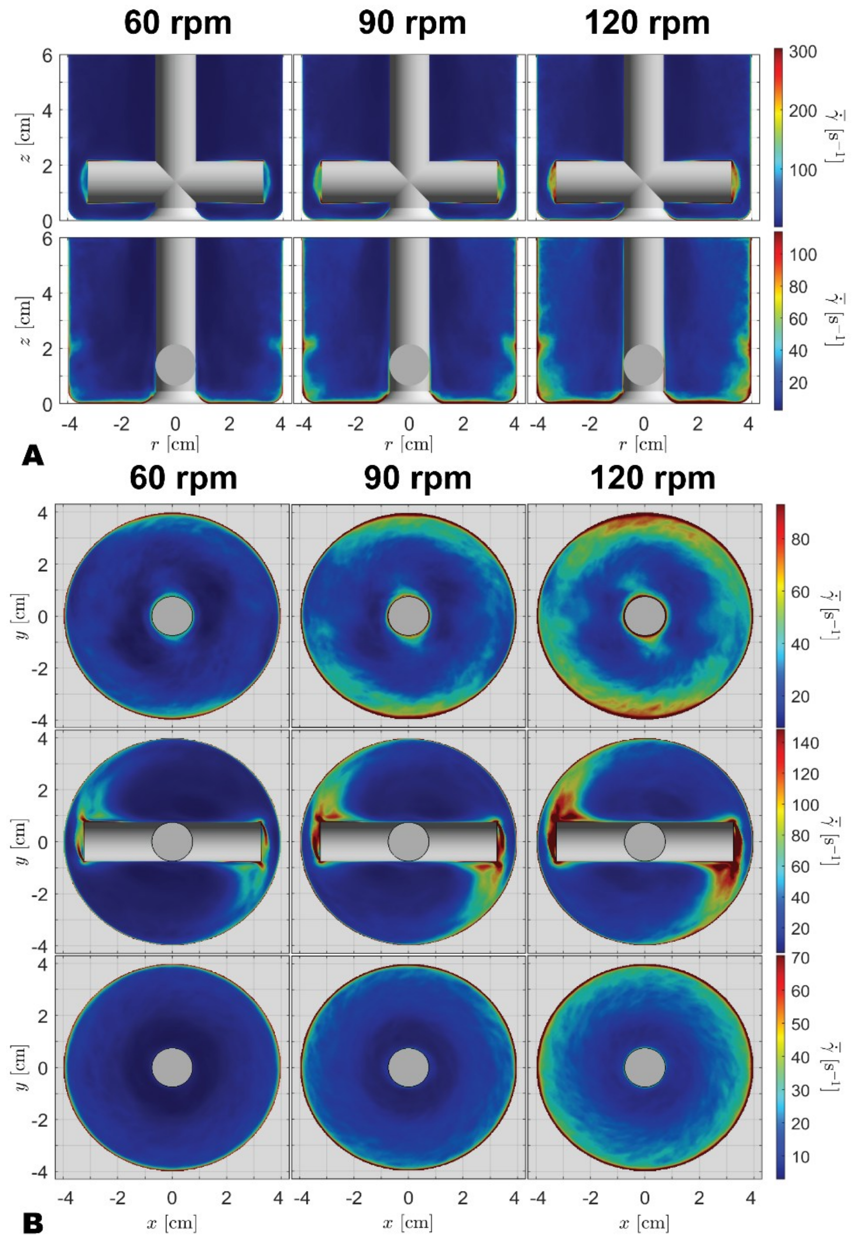

Figure S1: CFD simulation of spinner flask at 60, 90 and 120 rpm stirring rate, with the average shear strain rate at the plane of rotating impeller. A) Longitudinal view. **B)** Planar view.

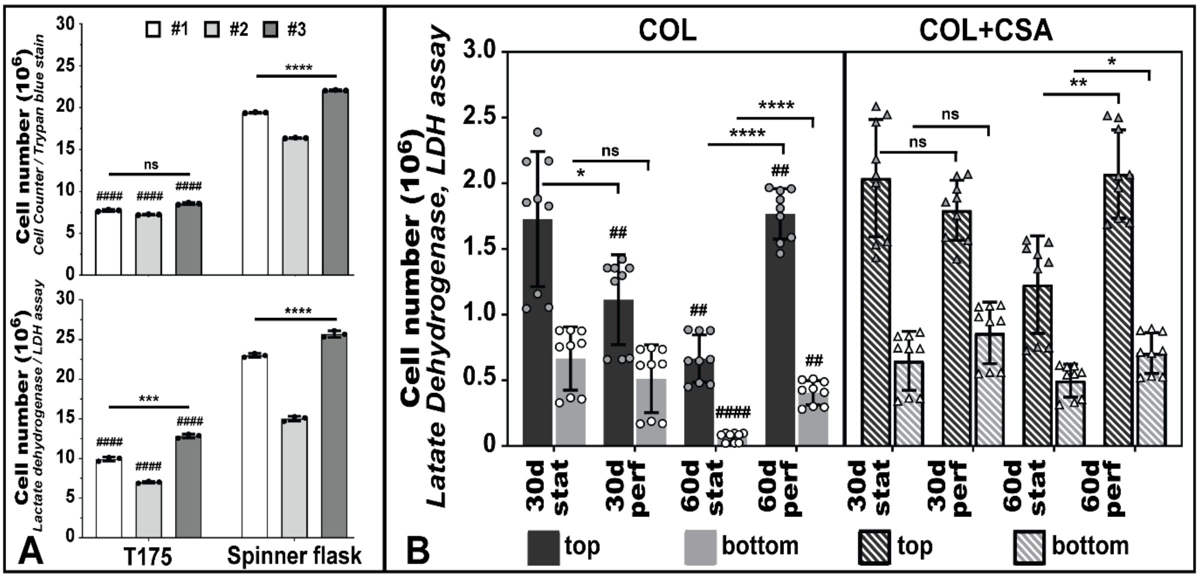

Figure S2: Overview of cell viability measurements. (A) Cell numbers derived from 2x T175 versus 1x Spinner flask after 12 days of cell expansion protocol. #1, #2, #3 indicate different hMSC donors (n=3). Top: cell counts from the automatic cell counter, viable hMSC were stained with Trypan Blue. Bottom: Lactate dehydrogenase (LDH assays) for cell viability assessments. Statistic: Student t-test T175 versus. Spinner flask variance within each donor. # $p < 0.05$, ## $p < 0.01$, ### $p < 0.001$, #### $p < 0.0001$; One-way ANOVA for variance between donors. * $p < 0.05$, ** $p < 0.01$, *** $p < 0.001$, **** $p < 0.0001$. **(B)** Cell numbers in respective bone constructs generated in static and perfusion setups on 30d and 60d. This LDH assay for cell viability is a corresponding assessment of the Absolute Cell Number determined from DNA concentrations, as shown in Figure 3. Statistic: Student t-test COL samples against corresponding COL+CSA samples (n=3, 3 independent experiments). # $p < 0.05$, ## $p < 0.01$, ### $p < 0.001$, #### $p < 0.0001$; Two-way ANOVA and Tukey Multiple t comparison (n=3, 3 independent experiments) within each scaffold type. * $p < 0.05$, ** $p < 0.01$, *** $p < 0.001$, **** $p < 0.0001$.

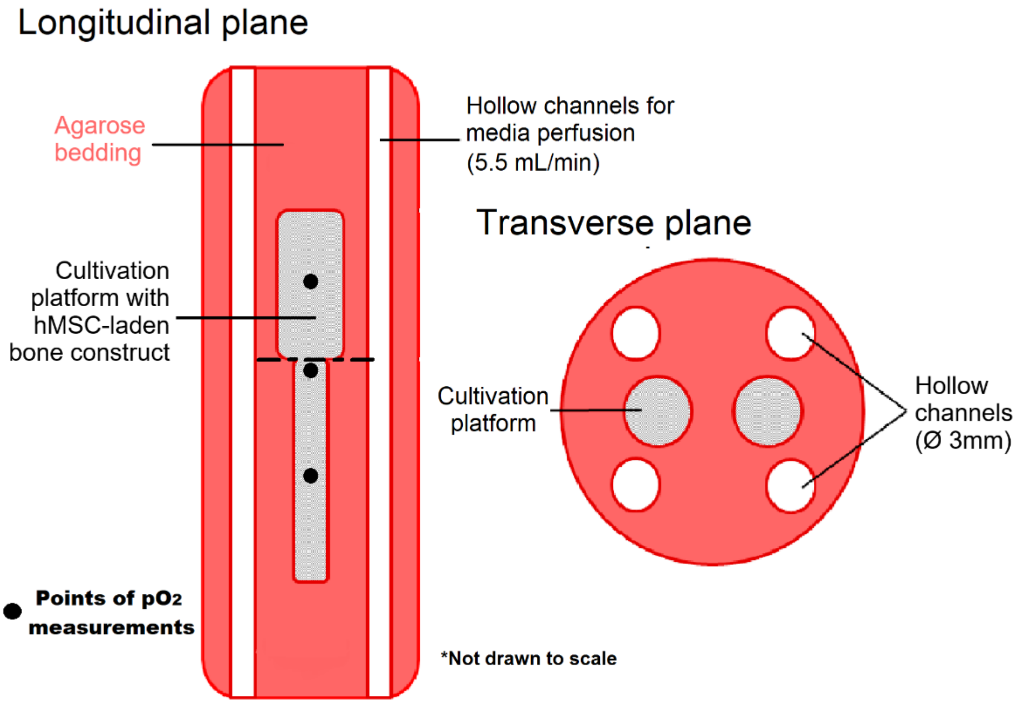

Figure S3: Overview of bioreactor setup and arrangement of hollow channels for medium perfusion. There are two compartments for hMSC-laden scaffolds in each perfusion bioreactor, and 2x 3 mm hollow channels for medium perfusion run along each compartment to support efficient mass transfer.

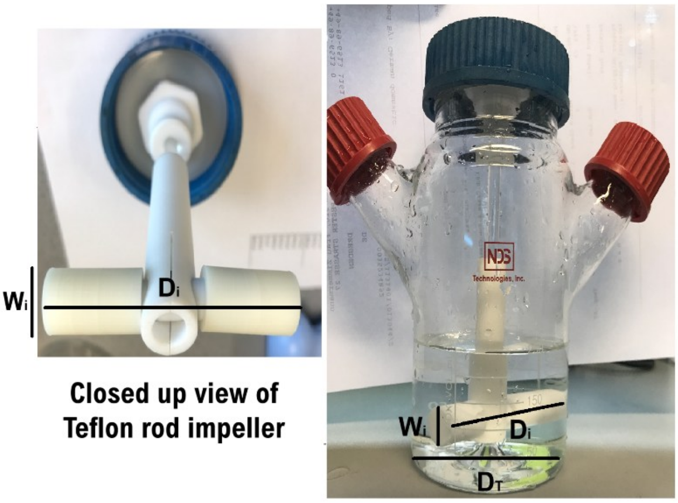

	Specifications applied in this study
Parameters	Values (units) 250 mL flask, rod impeller
ρ	1006 (kg/m^3)
υ	8.45 x 10^{-7} (m^2/s)
V_L	0.00025 (m^3)
D_i	0.065 (m)
D_T	0.085 (m)
w_i	0.015 (m)

Figure S4: Spinner flask specifications and impeller geometry. The specifications and measurements pertaining to the calculation of shear force in the spinner flasks are - medium density of DMEM (ρ), kinematic velocity (ν), total volume (V_L), impeller diameter (D_i), vessel diameter (D_T), and impeller width (w_i).

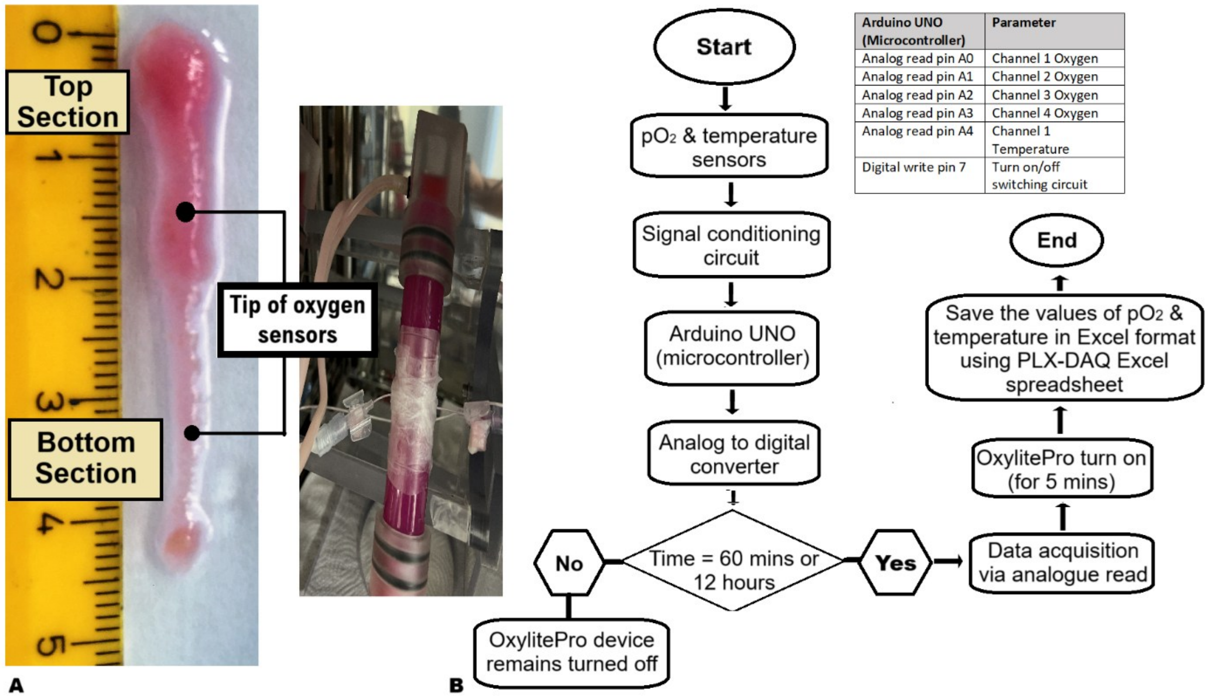

Figure S5: Overview of setup for oxygen tension (pO_2) measurements. (A) Position of sensor tip at the top and bottom sections of hMSC-laden construct (left), positioning of pO_2 sensors via 20G catheters. (**B**) Flow process of customized Arduino UNO data acquisition (DAQ) system connected to the OxylitePro to record measurements in real-time. The connections of Arduino pins to the BNC connectors are listed in the Table.
